# The pharmacology and mechanism of action of *Monascus purpureus* Went: a scoping review

**DOI:** 10.3389/fphar.2025.1600460

**Published:** 2025-07-30

**Authors:** Sitong Liu, Yaxin Xu, Jinhan Xie, Jing Hu, Yuetong Wang, Junhua Zhang, Myeong Soo Lee, Haiyin Hu, Lin Ang, Zhaochen Ji

**Affiliations:** ^1^ Tianjin University of Traditional Chinese Medicine, Tianjin, China; ^2^ Haihe Laboratory of Modern Chinese Medicine, Tianjin, China; ^3^ KM Science Research Division, Korea Institute of Oriental Medicine, Daejeon, Republic of Korea

**Keywords:** *Monascus purpureus* Went, pharmacological effects, pharmacological mechanisms, scope review, evidence

## Abstract

**Objective:**

The aim of this study is to review the recent studies on the pharmacology and mechanism of action of *Monascus purpureus* Went, analyze its medicinal value, and explore future research directions.

**Method:**

A scoping review was conducted by searching the China National Knowledge Infrastructure (CNKI), Wanfang database, VIP database, SinoMed, and PubMed from inception until September 2024. The basic information of the included studies, such as study types, disease types, main components, outcomes, and efficacy, was reviewed and summarized. Methodological quality was assessed using the SYRCLE’s risk of bias assessment tool for animal studies and the Cochrane risk of bias assessment tool for clinical trials.

**Results:**

We identified 251 studies from the five databases. Among them, 153 were experimental studies, 70 were reviews, and 28 were clinical trials. Of the experimental studies, molecular studies accounted for the largest portion, totaling 80 (52%). Among the reviews, research progress accounted for the most, totaling 41 (59%). The clinical trials studied the effects of *Monascus purpureus* Went and its related Chinese patent medicines and preparations. Of these, 17 (61%) used *Monascus purpureus* Went*-*related Chinese patent medicines and preparations as interventions and 11 (39%) used traditional Chinese medicine (TCM) formulations of *Monascus purpureus* Went as interventions. In terms of methodological quality, both animal studies and clinical trials related to *Monascus purpureus* Went showed deficiencies in randomized allocation sequence generation, allocation concealment, and blinding methods.

**Conclusion:**

We summarized existing studies on the active ingredients and effects of *Monascus purpureus* Went and found that it is necessary to improve the generation of random allocation sequences and the application of the blinding method in *Monascus purpureus* Went-related animal studies and clinical trials. When similar studies are conducted in the future, the specific methods of random assignment should be more clearly described, and blinding methods should be applied to improve the objectivity and accuracy of the studies, thereby providing a reference for selecting future research directions and establishing supporting evidence.

## 1 Introduction

*Monascus purpureus* Went is a traditional Chinese medicine (TCM) with a long history, produced through the fermentation of ordinary rice using Monascus species (Chen et al., 2000). It is mainly produced in Fujian, Zhejiang, and Jiangxi. Additionally, *Monascus purpureus* Went is sweet in taste, warm in nature, and belongs to the liver, spleen, stomach, and large intestine meridians, according to TCM theory. It is recorded in *the Supplement to Augmented Materia Medica* that *Monascus purpureus* Went has the effects of “promoting blood circulation and helping digestion, strengthening the spleen and warming the stomach, treating dysentery, and bringing down water.” Clinically, *Monascus purpureus* Went is mainly used to treat postpartum lochia, abdominal pain with stagnation, food accumulation and fullness, dysentery, and bruises (Song et al., 1999a).

In 2024, Japan’s Kobayashi Pharmaceutical Co., Ltd. experienced a safety incident involving “health products containing *Monascus purpureus* Went ingredients.” After investigation, the issue was potentially caused by contamination of the *Monascus purpureus* Went fermentation raw materials with penicillic acid or by inadequate cleanliness in the production environment, lead to the presence of penicillium mixed. This incident once again brought the efficacy and safety of red yeast into the global social hot spot (Ma 2025; Sun, 2025; Zhu et al., 2025).

Modern pharmacology has discovered that *Monascus purpureus* Went has lipid-lowering, anti-tumor, antioxidant, anti-osteoporosis, antibacterial, and other effects (Wei et al., 2023). In addition, *Monascus purpureus* Went has a wide range of applications in brewing, fermented foods, food coloring, and other fields. In recent years, new application fields have been gradually developed, such as animal husbandry and veterinary medicine, feed fermentation, healthy fermented foods, healthy drinks, and healthy seasonings (Xie, 1996). In recent years, the extraction of the lipid-lowering component *lovastatin* from *Monascus purpureus* Went (Endo, 1980) has further enhanced the research value of *Monascus purpureus* Went, attracting great attention from scholars both at home and abroad. The significant lipid-lowering effect and medicinal potential of *lovastatin* have inspired many scholars to conduct studies on the pharmacology and mechanism of action of this new component in *Monascus purpureus* Went, resulting in many remarkable findings, such as studying the lipid-lowering mechanism and content determination method of *lovastatin* (Wen et al., 2001), optimizing the extraction process of *lovastatin* from *Monascus purpureus* Went (Wang et al., 2024a), and producing drugs mainly composed of *lovastatin*, such as Xuezhikang (Kong et al., 2005). At present, the pharmacological research on *Monascus purpureus* Went still needs to be improved to further understand and develop its medicinal value and expand its application range.

A large number of studies on *Monascus purpureus* Went have been published, including reviews (Wang et al., 2024b). However, these published reviews lacked a comprehensive literature search, which led to limited references and unreliable evidence evaluation. Such limitations may introduce certain biases into the review, which is not conducive to the reference of other studies. In contrast, this study conducts a scoping review based on evidence-based medicine methods to systematically summarize the recent studies on the pharmacology and mechanism of action of *Monascus purpureus* Went, analyze its medicinal value, and explore future research directions.

## 2 Data and methods

### 2.1 Search strategy

Five databases, namely, China National Knowledge Infrastructure (CNKI), Wanfang database, VIP database, SinoMed, and PubMed, were searched from inception to September 2024. The search terms consisted of *Monascus purpureus* Went, Monascus, *Monascus purpureus* Went, pharmacology, pharmacological effect, pharmacological mechanism, biological activity, and active ingredient. The full search terms of all databases are shown in Supplementary Add S1.

### 2.2 Inclusion and exclusion criteria

Inclusion criteria: Studies focused on *Monascus purpureus* Went; study types were not limited, and the language was English or Chinese.

Exclusion criteria: Manuscripts with unavailable full text and duplicate publications were excluded. News reports, conference papers, or dissertations were also excluded.

### 2.3 Study screening and data extraction

The screening and extraction process was as follows: 1) NoteExpress software was used to exclude duplicate studies, 2) two reviewers performed an initial screening after reading the titles and abstracts based on the inclusion and exclusion criteria, 3) the full text was reviewed when additional information was needed for screening, and 4) any disagreements were resolved through discussion with a third researcher.

The basic information in the included studies, such as title, authors, year of publication, type of study, subject of study, study population, study method, intervention, duration of treatment, dosage, control measure, measurement index, method of measurement, result, and conclusion, was extracted.

### 2.4 Data analysis

We analyzed all extracted data fields, including bibliometric statistics, visual data analysis, and evidence graph analysis. We also systematically organized and comprehensively summarized study evidence information.

### 2.5 Quality assessment

Two reviewers assessed the methodological quality of the included studies, including animal studies and clinical trials, as this is an integral part of evidence-based research. The SYRCLE’s risk of bias assessment tool was used to assess the quality of animal studies, and the Cochrane risk of bias assessment tool was used to assess the quality of randomized controlled trials (RCTs). The evaluation results were indicated as “low risk,” “high risk,” or “unclear risk.”

## 3 Results

### 3.1 Search results

A total of 2,494 studies were initially searched. Among them, 970 were duplicates, and 1,223 were excluded after reading the title or abstract. Among 301 studies assessed in full text, 50 were excluded for the following reasons: not focusing on *Monascus purpureus* Went (n = 41) and lack of full text (n = 9). Finally, 251 studies were included in the final review.

**FIGURE 1:**
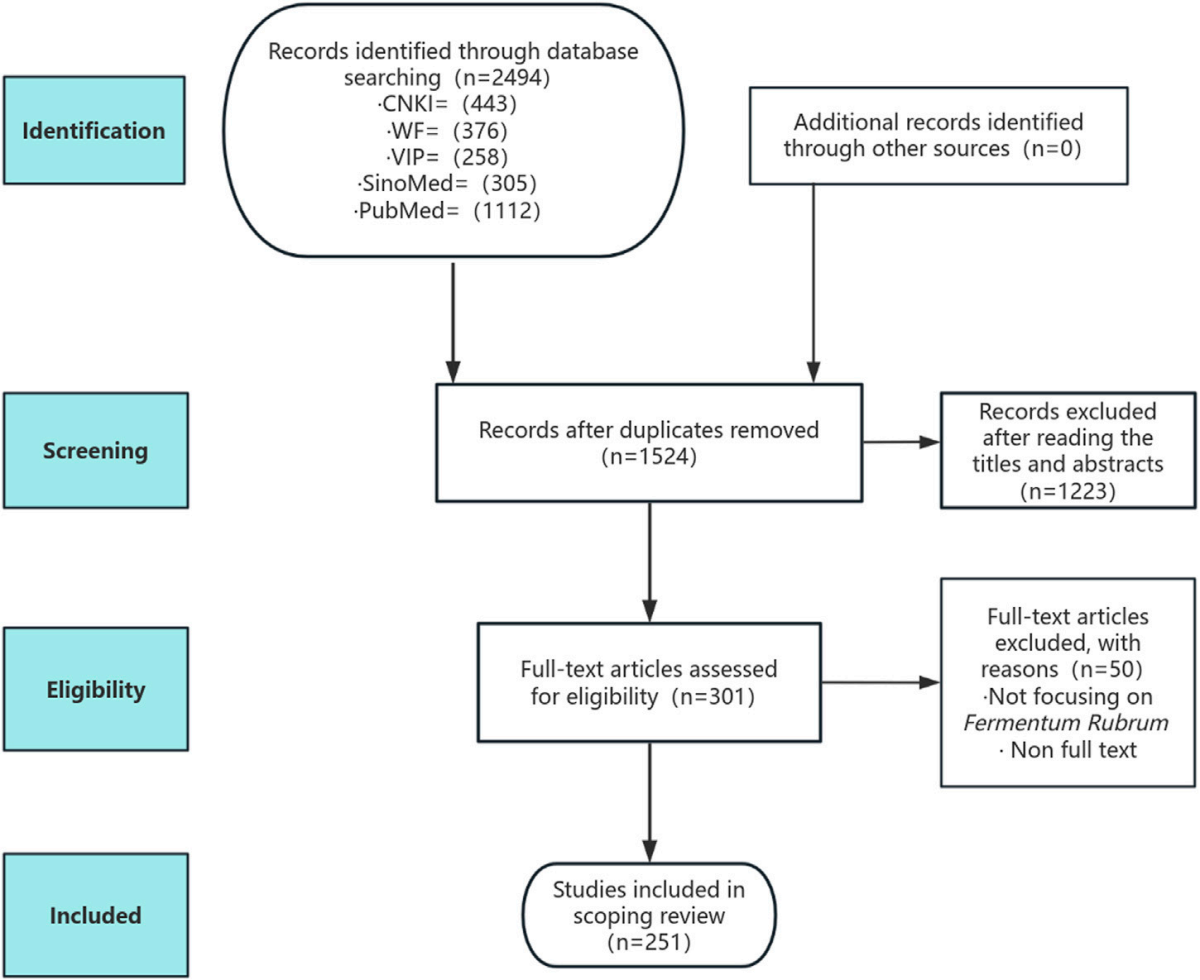
Flowchart of study screening.

The study screening process is presented in Figure 1.

### 3.2 Characteristics of included studies

**FIGURE 2:**
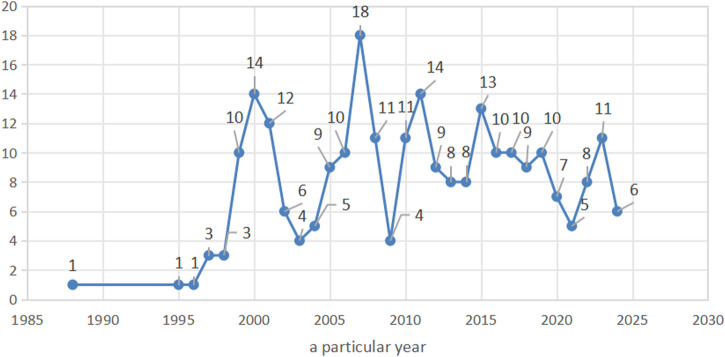
Number of studies related to *Monascus purpureus* Went each year.

A total of 251 literature studies were included, comprising 242 Chinese publications (96%) and 9 English publications (4%). There were 153 experimental studies (61%), 70 reviews (28%), and 28 clinical trials (11%). The included studies were published between 1988 and 2024, with the highest number published in 2007 (n = 18). The number of studies published each year is presented in Figure 2.

**FIGURE 3:**
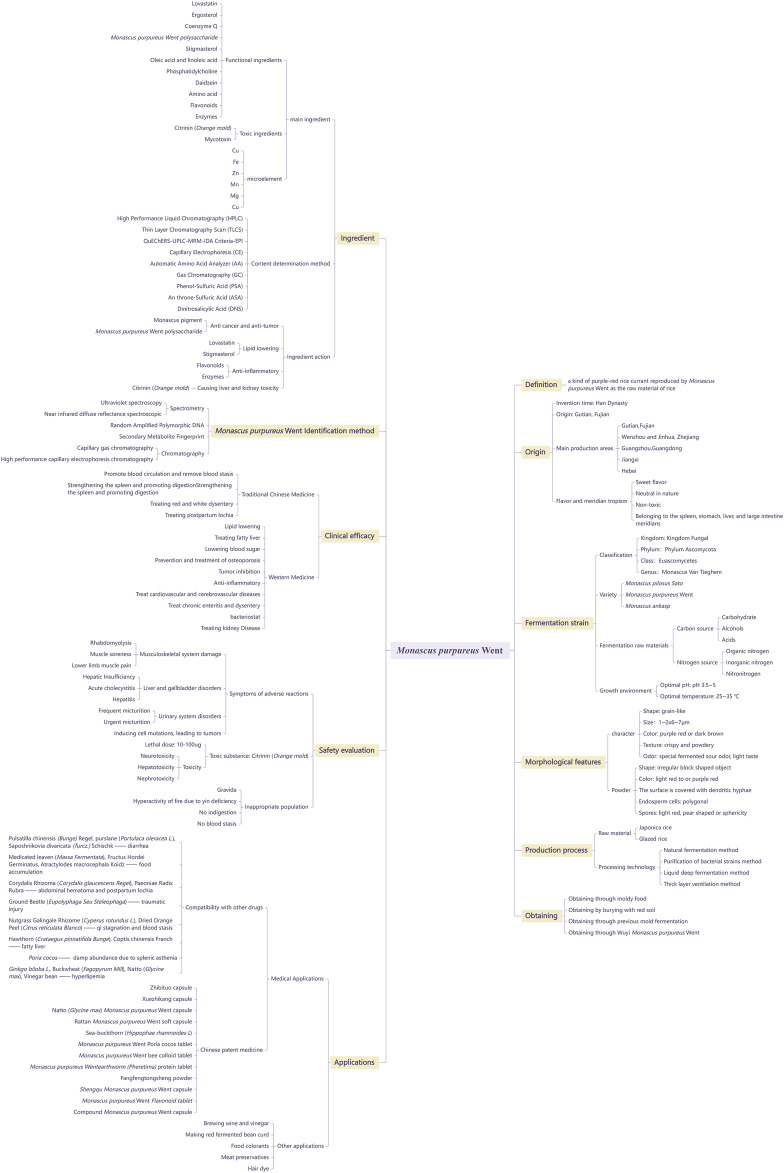
Chart of evidence from studies on *Monascus purpureus* Went.

We summarized and generalized evidence from studies on *Monascus purpureus* Went, including its definition, origin, fermentation strain, morphological features, production process, acquisition methods, ingredients, identification methods, clinical efficacy, safety evaluation, and applications. The chart of evidence from studies on *Monascus purpureus* Went is presented in Figure 3.

Among the experimental studies, molecular studies accounted for most of them, totaling 80 (52%). Among the reviews, research progress accounted for most of the studies, totaling 41 (59%). Clinical trials studied the effects of *Monascus purpureus* Went and its related Chinese patent medicine in the treatment of diseases, with 17 (61%) using *Monascus purpureus* Went-related Chinese patent medicines and preparations as interventions and 11 (39%) using TCM formulations of *Monascus purpureus* Went as interventions. The details of the research topics of the included studies are shown in Table 1.

**TABLE 1:** Study topics.

Study	Type	Study topic
Experimental studies	Molecular study (Wu and Luo, 2013)	Content determination (Luo et al., 2010) Chemical property (Wang et al., 2000) *Monascus purpureus* Went identification method (Yan, 1999) Component extraction process (Endo, 1980) Strain identification and screening (Zhu et al., 2025)
	Animal study (Huang et al., 2019)	Lipid lowering (Lu et al., 2006) Effect on bones (Wang et al., 2024b) Improvement of fatty liver (Zhu et al., 2025) Lowering blood pressure (Sun, 2025) Anti-inflammatory effect (Ma, 2025) Safety evaluation (Ma, 2025) Tumor inhibition (Song et al., 1999a) Cerebrovascular disease (Chen et al., 2000) Regulation of gastrointestinal diseases (Chen et al., 2000) Kidney disease (Chen et al., 2000)
	Cell study (Yu et al., 2000)	Identification and screening of bacterial strains (Xie, 1996) Efficacy (Wei et al., 2023) Physiological morphology of bacterial strains (Song et al., 1999a)
Reviews	Research progress (Luo and Zhang, 2011)	Pharmacological mechanisms (He, 2004) Active ingredients (Wang et al., 2024b) Clinical efficacy (Wen et al., 2001) Classification criteria (Chen et al., 2000)
	Popular science (Chen et al., 2005)	Pharmacological mechanisms (Wang et al., 2024a) *Monascus purpureus* Went-related food and drug use (Wei et al., 2023) Origin (Sun, 2025) Safety evaluation (Song et al., 1999a)
	Other (Zhu et al.)	Production (Song et al., 1999a) Industry standard (Song et al., 1999a) Selective breeding (Chen et al., 2000)
	Meta-analysis/SR (Song et al., 1999a)	Clinical efficacy (Chen et al., 2000) Safety evaluation (Chen et al., 2000)
Clinical trials	Randomized controlled trial (Chen et al., 2005)	TCM *Monascus purpureus* Went (Xie, 1996) Xuezhikang capsule (Wei et al., 2023) Shengqu *Monascus purpureus* Went capsule (Song et al., 1999a) Lipid-lowering *Monascus purpureus* Went micro-powder (Song et al., 1999a) Zhibituo capsule (Chen et al., 2000) Compound *Monascus purpureus* Went capsule (Chen et al., 2000) *Monascus purpureus* Went flavonoid tablet (Chen et al., 2000) Rattan *Monascus purpureus* Went soft capsule (Chen et al., 2000) *Coptis chinensis* Franch. *Monascus purpureus* Went medicine (Chen et al., 2000)
	Controlled trial (Wei et al., 2023)	TCM *Monascus purpureus* Went (Song et al., 1999a) *Monascus purpureus* Went compound preparation (Chen et al., 2000) Zhibituo capsule (Chen et al., 2000) *Monascus purpureus* Went capsule for reducing sugar (Chen et al., 2000) Danxi *Monascus purpureus* Went wine (Chen et al., 2000)

### 3.3 Experimental study

#### 3.3.1 Molecular study

Eighty molecular studies related to *Monascus purpureus* Went were included.

##### 3.3.1.1 Content determination

Forty studies determined the content of active ingredients in *Monascus purpureus* Went, with *lovastatin* and citrinin (*orange mold*) being the most common ingredients. The methods for content determination included high-performance liquid chromatography (HPLC) (n = 29), thin-layer chromatography scan (TLCS) (n = 2), capillary electrophoresis (CE) (n = 2), automatic amino acid analyzer (AA) (n = 2), gas chromatography (GC) (n = 1), quick, easy, cheap, effective, rugged, safe–ultra-performance liquid chromatography–multiple reaction monitoring–ion-dependent acquisition–criteria-enhanced product ion (QuEChERS-UPLC-MRM-IDA-Criteria-EPI) (n = 1), phenol–sulfuric acid (PSA) (n = 1), anthrone–sulfuric acid (ASA) (n = 1), dinitrosalicylic acid (DNS) (n = 1), flame atomic absorption spectroscopy (FAAS) (n = 1), and statistical analyses including statistical package for the social sciences principal component analysis and cluster analysis (SPSS PCA and SPSS CA) (n = 1).

##### 3.3.1.2 Chemical property

Fourteen studies examined the chemical properties of the active ingredients in *Monascus purpureus* Went, including antioxidant activity (n = 5), chemical structure (n = 5), lipid-lowering activity (n = 2), poisoning mechanism (n = 1), and protease and amylase activities (n = 1).

##### 3.3.1.3 *Monascus purpureus* Went identification method

Thirteen studies examined the methods for identifying different *Monascus purpureus* Went strains using chromatography (n = 6), spectroscopy (n = 4), random amplified polymorphic DNA (RAPD) (n = 2), and secondary metabolite fingerprint (SMF) (n = 1).

##### 3.3.1.4 Component extraction process

Eight studies investigated the extraction process of active ingredients in *Monascus purpureus* Went.

(1) *Lovastatin*: One study used 88% ethanol in an amount 13 times that of the raw material, with reflux extraction for 1.3 hours (n = 1); another study used six times the amount of 95% ethanol, with reflux extraction performed twice, each for 0.5 hours (n = 1); and a third study used 250 mL of ethanol, with reflux for 5 hours (n = 1).
(2) Monacolin K: One study used 70% ethanol (pH 7.5) with a material-to-solvent ratio of 1:30, an extraction time of 1.5 hours, and an extraction temperature of 50°C (n = 1); another study used pure methanol with an extraction temperature 60°C, a liquid-to-solid ratio of 20:1, and ultrasonic extraction for 1 hour (n = 1); and a third used 65% ethanol with a material-to-liquid ratio of 1:20, an extraction temperature of 70°C, and an extraction time of 3 hours (n = 1).
(3) Total flavonoid: One study used analytically pure ethanol and ultrasonic (50 kHz, 250 W) extraction for 20 min, followed by adsorption using 1 g of polyamide powder. The sample was then transferred to a chromatography column (inner diameter 1.0 cm) and eluted with methanol elution (0.5 mL/min) (n = 1).
(4) *Monascus purpureus* Went pigment: One study used a 70% ethanol aqueous solution with an extraction temperature of 60°C and an extraction time of 2 hours (n = 1).

##### 3.3.1.5 Strain identification and screening

Five studies examined the identification and screening of *Monascus purpureus* Went strains based on molecular biology.

#### 3.3.2 Animal study

Among the 58 animal studies, 54 used rat models (93%), 3 used rabbit models, and 1 used the quail model. A total of 55 studies determined the efficacy of *Monascus purpureus* Went, including double-armed studies (n = 6), three-armed studies (n = 10), and multi-armed studies (n = 39). Three studies determined the safety of erythromycin, including a two-armed study (n = 1), a three-armed study (n = 1), and a multi-armed study (n = 1). Fifty-four studies contained a blank control, and four studies did not contain a blank control.

Among them, 26 studies showed that *Monascus purpureus* Went had lipid lowering effects, involving TCM *Monascus purpureus* Went (n = 5), *Monascus purpureus* Went compound preparation (n = 3), vinegar bean lipid-lowering capsule (n = 1), *Monascus purpureus* Went earthworm (*Pheretima*) protein tablet (n = 1), *Monascus purpureus* Went *Allium sativum* L. fermentation extract (n = 1), *Monascus purpureus* Went *Poria cocos* (Schw.) Wolf tablet (n = 1), compounded *Monascus purpureus* Went capsule (n = 1), a mixture of *Monascus purpureus* Went and grape seed anthocyanidin (n = 1), *Monascus purpureus* Went bee glue tablet (n = 1), Xuezhikang capsule (n = 1), *lovastatin* (n = 1), natto (*Glycine max*) *Monascus purpureus* Went (n = 1), *Ginkgo biloba* L. *Monascus purpureus* Went vitamin grouping (n = 1), *Monascus purpureus* Went combined with Fang Feng Tong Sheng powder (n = 1), *Fagopyrum esculentum* Moench *Monascus purpureus* Went powder (n = 1), *Monascus purpureus* Went–phytosterol ester compound preparation (n = 1), Yunnan *Monascus purpureus* Went powder (n = 1), compounded *Monascus purpureus* Went extract (n = 1), yellow Monascus pigment (n = 1), and sea-buckthorn (*Hippophae rhamnoides* L.) *Monascus purpureus* Went capsule (n = 1). Twelve studies showed that *Monascus purpureus* Went had an effect on repairing bones, involving TCM *Monascus purpureus* Went (n = 11) and *Monascus purpureus* Went capsule-containing coenzyme Q10 (n = 1); five studies showed that *Monascus purpureus* Went had an effect on improving fatty liver, involving TCM *Monascus purpureus* Went (n = 3), *Monascus purpureus* Went *Crataegus pinnatifida* Bunge (n = 1), and *Coptis chinensis* Franch. *Monascus purpureus* Went medicine (n = 1); four studies showed that *Monascus purpureus* Went had an effect on lowering blood pressure, involving TCM *Monascus purpureus* Went (n = 4); three studies showed that *Monascus purpureus* Went had an effect on anti-inflammation, involving TCM *Monascus purpureus* Went (n = 3); three studies showed a good safety profile of *Monascus purpureus* Went, involving *Monascus purpureus* Went polysaccharide (n = 1), *Monascus purpureus* Went extract (n = 1), and *Panax Notoginseng* (Burk.) F.H.Chen *Monascus purpureus* Went compound preparation (n = 1); two studies showed that *Monascus purpureus* Went had an effect on tumor inhibition, involving *Monascus purpureus* Went polysaccharide (n = 2); one study showed that *Monascus purpureus* Went had an effect on cerebrovascular disease, involving TCM *Monascus purpureus* Went (n = 1); one study showed that *Monascus purpureus* Went had an effect on regulating the gastrointestinal tract, involving TCM *Monascus purpureus* Went (n = 1); and one study showed that *Monascus purpureus* Went had an effect on treating renal disease, involving *Monascus purpureus* Went extract (n = 1).

Among these studies, the most commonly used dose was 0.625 g/mL of aqueous *Monascus purpureus* Went solution, administered via irrigation at 10 mL/kg, and the most commonly used course of treatment was 28 days. The specific information on animal studies is presented in Table 2.

**TABLE 2:** Information on animal studies related to *Monascus purpureus* Went.

Inclusion of studies	Disease	Model	Method of administration	Intervention	Dosage	Course of treatment	Outcome indicator
Yan (1999)	Hyperlipemia	Rat	Irrigation	Distilled water vs. vinegar lipid-lowering capsule low dose (Chinese patent medicine) vs. vinegar lipid-lowering capsule medium dose (Chinese patent medicine) vs. vinegar lipid-lowering capsule high dose (Chinese patent medicine)	0.3 g/kg.bw, 0.6 g/kg.bw, and 1.2 g/kg.bw	28 days	①②③
Wang et al. (2000)	Nephrosis	Rat	Irrigation	TCM *Monascus purpureus* Went vs. blank control	0.8 g·kg^−1^·day^−1^	20 days	①②③④⑥⑦
Yu et al. (2000)	Hyperlipemia	Rabbit	Irrigation	Yunnan *Monascus purpureus* Went powder low dose (traditional TCM preparation) vs. Yunnan *Monascus purpureus* Went powder medium dose (traditional TCM preparation) vs. Yunnan *Monascus purpureus* Went powder high dose (traditional TCM preparation) vs. *lovastatin*	4 mg·kg^−1^·day^−1^, 6 mg·kg^−1^·day^−1^, 10 mg·kg^−1^·day^−1^, and 6 mg·kg^−1^·day^−1^	42 days	①②③④⑥⑦
Sun et al. (2001a)	Hyperlipemia	Rat	Irrigation	TCM *Monascus purpureus* Went low dose vs. TCM *Monascus purpureus* Went high dose vs. blank control	0.6 g/kg BW and 1.2 g/kg BW	28 days	①②③
Sun et al. (2001b)	Hyperlipemia	Rat	Irrigation	TCM *Monascus purpureus* Went low dose vs. TCM *Monascus purpureus* Went medium dose vs. TCM *Monascus purpureus* Went high dose vs. blank control	0.4 g/kg BW, 0.8 g/kg BW, and 1.2 g/kg BW	21 days	⑧
Wang et al. (2002)	Fatty liver	Quail	Irrigation	TCM *Monascus purpureus* Went low dose vs. TCM *Monascus purpureus* Went high dose vs. Dongbao liver-healthy vs. *lovastatin* vs. blank control	0.8 g·kg^−1^, 1.6 g·kg^−1^, and 0.6 g·kg^−1^	20 days	①②③④
He (2004)	Hyperlipemia and atherosclerotic lesions	Rabbit	Irrigation	*Monascus purpureus* Went *Allium sativum* L. fermentation extract vs. blank control	100 g/day	126 days	①②③④
Lu et al. (2004)	Osteoporosis	Rat	Irrigation	TCM *Monascus purpureus* Went vs. α-D3 vs. blank control	10 mL/kg	98 days	⑨⑩⑪⑰
Tang et al. (2004)	Hypertension	Rat	Irrigation	TCM *Monascus purpureus* Went vs. blank control	0.417 g/(kg·day)	21 days	⑫⑬
Chen et al. (2005)	Hyperlipemia	Rat	Irrigation	*Lovastatin* low dose vs. *lovastatin* medium dose vs. *lovastatin* high dose vs. *Gynostemma Blume*	5, 15, and 30 mg/kg and 20 mg/kg	42 days	④⑭
Lu et al. (2005a)	Osteoporosis	Rat	Irrigation	TCM *Monascus purpureus* Went vs. α-D3 vs. blank control	10 mL/kg	90 days	⑮⑯
Lu et al. (2005b)	Osteoporosis	Rat	Irrigation	TCM *Monascus purpureus* Went vs. α -D3 vs. blank control	10 mL/kg	84 days	⑪⑰
Wang et al. (2005)	Hyperlipemia	Rat	Irrigation	Compound *Monascus purpureus* Went capsule high dose vs. compound *Monascus purpureus* Went capsule medium dose vs. compound *Monascus purpureus* Went capsule high dose vs. blank control	167, 333, and 1,000 mg/kg	28 days	①②③
Lu et al. (2006)	Osteoporosis	Rat	Irrigation	TCM *Monascus purpureus* Went vs. Pravastatin vs. Premarin vs. blank control	10 mL/kg	10 days	⑯⑱
Wang et al. (2006a)	Air pouch synovitis	Rat	Irrigation	Ibuprofen vs. *lovastatin* high dose vs. *lovastatin* low dose vs. TCM *Monascus purpureus* Went low dose vs. TCM *Monascus purpureus* Went medium dose vs. TCM *Monascus purpureus* Went high dose vs. blank control	30 mg/kg, 3.6 mg/kg, 1.8 kg/mg, 200 mg/kg, 100 mg/kg, and 50 mg/kg	5 days	⑲⑳㉑
Wang et al. (2006b)	Small ear swelling	Rat	Irrigation	TCM *Monascus purpureus* Went low dose vs. TCM *Monascus purpureus* Went medium dose vs. TCM *Monascus purpureus* Went high dose vs. *lovastatin* high dose vs. *lovastatin* low dose vs. blank control	300 mg/kg, 150 mg/kg, 75 mg/kg, 5.2 mg/kg, 2.6 mg/kg	5 days	㉒
Wu et al. (2006)	Osteoporosis	Rat	Irrigation	TCM *Monascus purpureus* Went vs. Pravastatin vs. Premarin vs. blank control	10 mL/kg	10 days	⑮
Ding (2007)	Tumor	Rat	Irrigation	*Monascus purpureus* Went polysaccharide low dose vs. *Monascus purpureus* Went polysaccharide medium dose vs. *Monascus purpureus* Went polysaccharide high dose vs. blank control	100, 400, and 800 mg/kg	14 days	㉓㉔
Lei et al. (2007)	Hypertension	Rat	Irrigation	TCM *Monascus purpureus* Went high dose vs. TCM *Monascus purpureus* Went low dose vs. positive vs. blank control	0.83 g/kg, 0.42 g/kg, and 10 mg/kg	28 days	⑫⑬㉕㉖
Wang et al. (2007)	Hyperlipemia	Rat	Irrigation	*Monascus purpureus* Went compound preparation high dose vs. *Monascus purpureus* Went compound preparation medium dose vs. *Monascus purpureus* Went compound preparation high dose vs. blank control	0.47 g/kg, 0.23 g/kg, and 0.12 g/kg	30 days	①②③
Wu et al. (2007a)	Fracture	Rat	Irrigation	TCM *Monascus purpureus* Went vs. bone-knitting tablet vs. blank control	10 mL/kg	42 days	⑮㉗
Wu et al. (2007b)	Fracture	Rat	Irrigation	TCM *Monascus purpureus* Went vs. blank control	10 mL/kg	42 days	㉘
Zheng et al. (2007)	Hypertension	Rat	Irrigation	TCM *Monascus purpureus* Went low dose vs. TCM *Monascus purpureus* Went medium dose vs. TCM *Monascus purpureus* Went high dose vs. captopril vs. indapamide vs. blank control	0.25 g/BW/day, 0.42 g/BW/day, 0.84 g/BW/day, 10 mg/BW/day, and 0.21 mg/BW/day	28 days	⑧⑫⑬㉕㉖
Jiang et al. (2008)	Hyperlipemia	Rat	Irrigation	Mixture of *Monascus purpureus* Went and grape seed *anthocyanidin* low dose vs. mixture of *Monascus purpureus* Went and grape seed *anthocyanidin* medium dose vs. mixture of *Monascus purpureus* Went and grape seed *anthocyanidin* high dose vs. blank control	12 mg/mL, 120 mg/mL, and 360 mg/mL	70 days	①②③⑤
Lu et al. (2008)	osteoporosis	Rat	Irrigation	TCM *Monascus purpureus* Went vs. pravastatin vs. Premarin vs. blank control	10 mL/kg	10 days	⑯⑱㉙
Wang et al. (2008)	Arthritis	Rat	Irrigation	TCM *Monascus purpureus* Went vs. indomethacin vs. blank control	500 mg·kg^−1^ and 5 mg·kg^−1^	34 days	㉚㉛㉜
Chen et al. (2010)	Hyperlipemia	Rat	Irrigation	Compounded *Monascus purpureus* Went extract low dose vs. compounded *Monascus purpureus* Went extract medium dose vs. compounded *Monascus purpureus* Went extract high dose vs. lovastatin vs. blank control	0.6, 1.2, and 2.4 g·kg^−1^ and 0.24 g·kg^−1^	21 days	①②③④
Luo et al. (2010)	Non-alcoholic fatty liver	Rat	Irrigation	TCM *Monascus purpureus* Went low dose vs. TCM *Monascus purpureus* Went medium dose vs. TCM *Monascus purpureus* Went high dose vs. *Gynostemma Blume* vs. blank control	1 g/(kg · day), 0.33 g/(kg · day), and 0.17 g/(kg · day)	56 days	㉟㊱㊲
Luo and Zhang (2011)	Non-alcoholic fatty liver	Rat	Irrigation	TCM *Monascus purpureus* Went low dose vs. TCM *Monascus purpureus* Went medium dose vs. TCM *Monascus purpureus* Went high dose vs. *Gynostemma Blume* vs. blank control	1 g/(kg · day), 0.33 g/(kg · day), and 0.17 g/(kg · day)	56 days	①②④㉝㉞
Zhao and Liu (2011)	Hyperlipemia	Rat	Irrigation	*Monascus purpureus* Went–phytosterol ester compound preparation low dose vs. *Monascus purpureus* Went–phytosterol ester compound preparation medium dose vs. *Monascus purpureus* Went–phytosterol ester compound preparation high dose vs. Xuezhikang capsules vs. blank control	0.167, 0.333, and 1.0 g/kg	45 days	①②③④
Zhou et al. (2011)	Tumor	Rat	Irrigation	*Monascus purpureus* Went polysaccharide low dose vs. *Monascus purpureus* Went polysaccharide medium dose vs. *Monascus purpureus* Went polysaccharide high dose vs. Tremella polysaccharide vs. blank control	200, 100, and 50 mg/(kg · day) and 50 mg/(kg · day)	14 days	㉓㉔㊳
Ou-yang et al. (2012)	Hyperlipemia	Rat	Irrigation	*Fagopyrum esculentum* Moench *Monascus purpureus* Went powder high dose vs. *Fagopyrum esculentum* Moench *Monascus purpureus* Went powder medium dose vs. *Fagopyrum esculentum* Moench *Monascus purpureus* Went powder small dose vs. Xuezhikang capsule vs. blank control	1,200, 600, and 300 mg/kg and 400 mg/kg	14 days	①②③④
Zhai et al. (2012)	Hyperlipemia	Rat	Irrigation	*Monascus purpureus* Went compound preparation low dose vs. *Monascus purpureus* Went compound preparation medium dose vs. *Monascus purpureus* Went compound preparation high dose vs. Xuezhikang capsule vs. blank control	0.21 g·kg^-1^BW, 0.42 g·kg-1BW, 1.25 g·kg^-1^BW, –0.2 g·kg^-1^BW	30 days	①②③④
Du and Chen (2013)	Safety evaluation	Rat	Irrigation	TCM *Monascus purpureus* Went vs. blank control	15 g/kg	14 days	㊴
Liu et al. (2013)	Hyperlipemia	Rat	Irrigation	Compounded *Monascus purpureus* Went capsule low dose vs. compounded *Monascus purpureus* Went capsule medium dose vs. compounded *Monascus purpureus* Went capsule high dose vs. blank control	167, 333, and 1,000 mg/kg	30 days	①②③
Lin et al. (2014)	Hyperlipemia	Rat	Irrigation	TCM *Monascus purpureus* Went vs. blank control	10 mL/kg	28 days	①②③④㊵㊶
Su et al. (2014)	Hyperlipemia	Rat	Irrigation	Xuezhikang capsule vs. blank control	500 mg·kg^−1^·day^−1^	14 days	㊷
Zhou (2014)	Hyperlipemia	Rat	Irrigation	TCM *Monascus purpureus* Went low dose vs. TCM *Monascus purpureus* Went medium dose vs. TCM *Monascus purpureus* Went high dose vs. blank control	0.5, 1.0, and 2.0 g/kg	28 days	①②③④
Gao et al. (2015)	Hyperlipemia	Rat	Irrigation	TCM *Monascus purpureus* Went combined with Fang Feng Tong Sheng powder (Chinese patent medicine) vs. Fang Feng Tong Sheng powder vs. blank control	1 mL/time and 2 times/day	30 days	①②③④
Qian et al. (2015)	Osteoporosis	Rat	Irrigation	TCM *Monascus purpureus* Went low dose vs. TCM *Monascus purpureus* Went medium dose vs. TCM *Monascus purpureus* Went high dose vs. *fluvastatin* vs. blank control	(0.1, 0.5, and 1.0 g/mL) 10 mL/kg and (0.1 g/L) 10 mL/kg	35 days	㊸
Lu et al. (2016)	Osteoporosis	Rat	Irrigation	*Monascus purpureus* Went capsule-containing coenzyme Q10 vs. diethylstilbestrol vs. blank control	0.5 tablet ·kg^−1^ and 30 μg·kg^−1^	60 days	⑰㊹
Lu et al. (2017)	Osteoporosis	Rat	Irrigation	TCM *Monascus purpureus* Went vs. estrogen vs. blank control	10 mL/kg	84 days	⑰㉙㉛
Pang et al. (2017)	Hyperlipemia	Rat	Irrigation	Natto (*Glycine max*) *Monascus purpureus* Went low dose vs. natto (*Glycine max*) *Monascus purpureus* Went medium dose vs. natto (*Glycine max*) *Monascus purpureus* Went high dose vs. blank control	0.2, 0.4, and 0.8 g/kg BW	30 days	①②③④
Zhang et al. (2017)	Safety evaluation	Rat	Irrigation	*Monascus purpureus* Went polysaccharide low dose vs. *Monascus purpureus* Went polysaccharide high dose vs. blank control	100 mg/(kg · day) and 300 mg/(kg · day)	21 days	㉔㉝㉞㉟㊴
Shen et al. (2018)	Osteoporosis	Rat	Irrigation	TCM *Monascus purpureus* Went vs. estrogen vs. blank control	10 mL/kg	56 days	⑪㊺
Zhou et al. (2018)	Hyperlipemia	rabbit	Irrigation	Sea-buckthorn (*Hippophae rhamnoides* L.) *Monascus purpureus* Went capsule high dose vs. sea-buckthorn (*Hippophae rhamnoides* L.) *Monascus purpureus* Went capsule low dose vs. simvastatin vs. blank control	3.6 g/kg, 1.8 g/kg, and 0.005 g/kg	15 days	①②③④
Huang et al. (2019)	Hyperlipemia	Rat	Irrigation	*Monascus purpureus* Went bee glue tablet low dose vs. *Monascus purpureus* Went bee glue tablet medium dose vs. *Monascus purpureus* Went bee glue tablet high dose vs. blank control	0.17, 0.33, and 1.00 g/kg	42 days	①②④
Liu et al. (2019)	Hyperlipemia	Rat	Irrigation	Yellow Monascus pigment low dose vs. yellow Monascus pigment medium dose vs. yellow Monascus pigment high dose vs. SIM vs. blank control	50, 100, and 200 mg/(kg · day) and 15 mg/(kg · day)	28 days	①②㉝㉞
Luo et al. (2019)	Syndrome of food retention due to spleen deficiency	Rat	Irrigation	TCM *Monascus purpureus* Went unleavened vs. TCM *Monascus purpureus* Went fermented vs. domperidone vs. blank control	1.17 g·kg^-1^·day^-1^, 1.17 g·kg^-1^·day^-1^, and 3.9 mg·kg^-1^·day^-1^	10 days	㊻
Wang (2019)	Hyperlipemia	Rat	Irrigation	TCM *Monascus purpureus* Went low dose vs. TCM *Monascus purpureus* Went medium dose vs. TCM *Monascus purpureus* Went high dose vs. blank control	5 mg/kg, 30 mg/kg, and 90 mg/kg	31 days	①②
Liu et al. (2020)	Hyperlipemia	Rat	Irrigation	*Monascus purpureus* Went *Poria cocos* (Schw.) Wolf tablet low dose vs. *Monascus purpureus* Went *Poria cocos* (Schw.) Wolf tablet medium dose vs. *Monascus purpureus* Went *Poria cocos* (Schw.) Wolf tablet high dose vs. Xuezhikang capsule vs. blank control	0.79 g/kg, 1.58 g/kg, 4.75 g/kg, and 0.2 g/kg	28 days	①②③④㊻
Liu et al. (2021)	Hyperlipemia	Rat	Irrigation	*Ginkgo biloba* L. *Monascus purpureus* Went vitamin grouping (low dose) vs. *Ginkgo biloba* L. *Monascus purpureus* Went vitamin grouping (high dose) vs. positive	25 mg/kg and 50 mg/kg	32 days	①②④
Sun et al. (2022)	Cerebral small vessel disease	Rat	Irrigation	TCM *Monascus purpureus* Went low dose vs. TCM *Monascus purpureus* Went medium dose vs. TCM *Monascus purpureus* Went high dose vs. nimodipine vs. blank control	0.75 g·kg^-1^, 1.5 g·kg^−1^, 3 g·kg^-1^, and 20 mg·kg^−1^	30 days	㊼
Ma et al. (2022)	Safety evaluation	Rat	Irrigation	*Panax Notoginseng* (Burk.) F.H.Chen and *Monascus purpureus* Went compound preparation high dose vs. *Panax Notoginseng* (Burk.) F.H.Chen and *Monascus purpureus* Went compound preparation medium dose vs. *Panax Notoginseng* (Burk.) F.H.Chen and *Monascus purpureus* Went compound preparation low dose vs. blank control	2, 4, and 8 g·(kg·day)^−1^ and 40 mg/kg	14 days	㊽
Zheng et al. (2022)	Hyperlipemia	Rat	Irrigation	TCM *Monascus purpureus* Went low dose vs. TCM *Monascus purpureus* Went medium dose vs. TCM *Monascus purpureus* Went high dose vs. *lovastatin* vs. blank control	0.09 mg/kg, 0.16 mg/kg, 0.21 mg/kg, and 0.21 mg/kg	42 days	①②③④㉝㉞
Ding and Ren (2023)	Hyperlipemia	Rat	Irrigation	*Monascus purpureus* Went earthworm (*Pheretima*) protein tablet low dose vs. *Monascus purpureus* Went earthworm (*Pheretima*) protein tablet medium dose vs. *Monascus purpureus* Went earthworm (*Pheretima*) protein tablet high dose vs. *Simvastatin* vs. blank control	0.167 g/kg, 0.333 g/kg, 0.666 g/kg, and 0.01 g/kg/d	28 days	①②③④
Yu et al. (2023)	Non alcoholic steatohepatitis	Rat	Irrigation	*Monascus purpureus* Went*–Crataegus pinnatifida* Bunge high dose vs. *Monascus purpureus* Went*–Crataegus pinnatifida* Bunge low dose vs. PPC vs. blank control	1.323 g·(kg·day)^-1^, 2.646 g·(kg·day)^-1^, and 0.086 g·(kg·day)^-1^	24 days	①②④㉛㉝㉞
Zhang et al. (2023)	Non-alcoholic fatty liver	Rat	Irrigation	*Coptis chinensis* Franch. *Monascus purpureus* Went medicine high dose vs. *Coptis chinensis* Franch. *Monascus purpureus* Went medicine low dose vs. obeticholic acid vs. blank control	2.1 g/kg, 1.1 g/kg, and 0.5 mg/kg	28 days	①②④⑤㉝㉞㉟㊲

Note: ①, total cholesterol (TC) level; ②, triglyceride (TG) level; ③, high-density lipoprotein cholesterol (HDL-C) level; ④, low-density lipoprotein cholesterol (LDL-C) level; ⑤, plasma oxygen-free radical (SOD, MDA, and GSH Px) level; ⑥, ApoA level; ⑦, ApoB level; ⑧, systolic blood pressure (SBP); ⑨, bone gla protein (BGP) level; ⑩, calcitonin (CT) level; ⑪, bone density; ⑫, endothelin (ET) level; ⑬, calcitonin gene-related peptide (CGRP) level; ⑭, atherosclerotic index (AI); ⑮, BMP-2 level; ⑯, osteoblast number; ⑰, bone biomechanical properties; ⑱, number of mineralized nodules; ⑲,white blood cell (WBC) count; ⑳, C-reactive protein (CRP) level; ㉑, malondialdehyde (MDA) level; ㉒, degree of swelling; ㉓, tumor suppression Rate; ㉔, organ index; ㉕, lung tissue ACE level; ㉖, aortic NOS level; ㉗, Nilsson’s histologic score; ㉘, bone tissue morphometric parameters; ㉙, ALP level; ㉚, arthritis index (AI) and pathology score; ㉛, serum TNF-α levels; ㉜, synovial MCP-1 and RANTES levels; ㉝, ALT levels; ㉞, AST levels; ㉟, blood glucose levels; ㊱, GIR levels; ㊲, insulin levels; ㊳, relative growth rate; ㊴, body weight and liver weight; ㊵, serum adiponectin levels; ㊶, AdipoR1/AdipoR2 mRNA expression levels; ㊷, EPC proliferation, adhesion, migration, and *in vitro* angiogenesis ability; ㊸, BMP-4 mRNA and protein expression levels in bone scab tissues; ㊹, bone calcium, bone phosphorus, and hydroxyproline content; ㊺, bone tissue RANKL, OPG protein, and mRNA expression levels; ㊻, serum levels of motilin (MTL), gastrin (GAS), 5-hydroxytryptamine (5-HT), and vasoactive intestinal peptide (VIP); ㊼, PI3K/AKT/mTOR protein expression levels; ㊽, cell micro-nucleus breakage, deletion, reciprocal translocation, circular chromosome, monovalent body, and cell aberration rate.

All 58 animal studies were randomized controlled studies. Among them, 55 studies only reported “randomization,” and 3 used the random number table method. A total of 58 studies reported that the experimental group was consistent with the control group at baseline. Fifty-seven studies did not report allocation concealment and blinding, and only one blinded the investigator and the animal keeper. Forty-two studies did not report whether the environments in which the animals were placed were randomized or not, and 16 studies reported that the animals were grown in the same environments; no missing data appeared. The risk of bias assessment of animal studies is presented in Table 3.

**TABLE 3:** Risk of bias assessment of animal studies.

Included in the study	①	②	③	④	⑤	⑥	⑦	⑧	⑨	⑩
Yan (1999)	Unclear	Low risk	Unclear	Unclear	Unclear	Unclear	Unclear	Low risk	Low risk	Unclear
Wang et al. (2000)	Unclear	Low risk	Unclear	Unclear	Unclear	Unclear	Unclear	Low risk	Low risk	High risk
Yu et al. (2000)	Unclear	Low risk	Unclear	Unclear	Unclear	Unclear	Unclear	Low risk	Low risk	Unclear
Sun et al. (2001a)	Unclear	Low risk	Unclear	Unclear	Unclear	Unclear	Unclear	Low risk	Low risk	Unclear
Sun et al. (2001b)	Unclear	Low risk	Unclear	Unclear	Unclear	Unclear	Unclear	Low risk	Low risk	Unclear
Wang et al. (2002)	Unclear	Low risk	Unclear	Unclear	Unclear	Unclear	Unclear	Low risk	Low risk	Unclear
He (2004)	Unclear	Low risk	Unclear	Unclear	Unclear	Unclear	Unclear	Low risk	Low risk	Unclear
Lu et al. (2004)	Unclear	Low risk	Unclear	Low risk	Unclear	Unclear	Unclear	Low risk	Low risk	Unclear
Tang et al. (2004)	Unclear	Low risk	Unclear	Unclear	Unclear	Unclear	Unclear	Low risk	Low risk	Unclear
Chen et al. (2005)	Unclear	Low risk	Unclear	Unclear	Unclear	Unclear	Unclear	Low risk	Low risk	High risk
Lu et al. (2005a)	Unclear	Low risk	Unclear	Low risk	Unclear	Unclear	Unclear	Low risk	Low risk	High risk
Lu et al. (2005b)	Unclear	Low risk	Unclear	Low risk	Unclear	Unclear	Unclear	Low risk	Low risk	High risk
Wang et al. (2005)	Unclear	Low risk	Unclear	Low risk	Unclear	Unclear	Unclear	Low risk	Low risk	Unclear
Lu et al. (2006)	Unclear	Low risk	Unclear	Low risk	Unclear	Unclear	Unclear	Low risk	Low risk	High risk
Wang et al. (2006a)	Unclear	Low risk	Unclear	Unclear	Unclear	Unclear	Unclear	Low risk	Low risk	Unclear
Wang et al. (2006b)	Unclear	Low risk	Unclear	Unclear	Unclear	Unclear	Unclear	Low risk	Low risk	Unclear
Wu et al. (2006)	Unclear	Low risk	Unclear	Low risk	Unclear	Unclear	Unclear	Low risk	Low risk	High risk
Ding (2007)	Unclear	Low risk	Unclear	Unclear	Unclear	Unclear	Unclear	Low risk	Low risk	High risk
Lei et al. (2007)	Unclear	Low risk	Unclear	Unclear	Unclear	Unclear	Unclear	Low risk	Low risk	Unclear
Wang et al. (2007)	Unclear	Low risk	Unclear	Unclear	Unclear	Unclear	Unclear	Low risk	Low risk	Unclear
Wu et al. (2007a)	Low risk (random digital table method)	Low risk	Unclear	Unclear	Unclear	Unclear	Unclear	Low risk	Low risk	High risk
Wu et al. (2007b)	Low risk (random digital table method)	Low risk	Unclear	Unclear	Unclear	Unclear	Unclear	Low risk	Low risk	High risk
Zheng et al. (2007)	Unclear	Low risk	Unclear	Unclear	Unclear	Unclear	Unclear	Low risk	Low risk	Unclear
Jiang et al. (2008)	Unclear	Low risk	Unclear	Low risk	Unclear	Unclear	Unclear	Low risk	Low risk	Unclear
Lu et al. (2008)	Unclear	Low risk	Unclear	Unclear	Unclear	Unclear	Unclear	Low risk	Low risk	Unclear
Wang et al. (2008)	Unclear	Low risk	Unclear	Unclear	Unclear	Unclear	Unclear	Low risk	Low risk	High risk
Chen et al. (2010)	Unclear	Low risk	Unclear	Unclear	Unclear	Unclear	Unclear	Low risk	Low risk	Unclear
Luo et al. (2010)	Unclear	Low risk	Unclear	Unclear	Unclear	Unclear	Unclear	Low risk	Low risk	Unclear
Luo and Zhang (2011)	Unclear	Low risk	Unclear	Unclear	Unclear	Unclear	Unclear	Low risk	Low risk	Unclear
Zhao and Liu (2011)	Unclear	Low risk	Unclear	Low risk	Unclear	Unclear	Unclear	Low risk	Low risk	Unclear
Zhou et al. (2011)	Unclear	Low risk	Unclear	Unclear	Unclear	Unclear	Unclear	Low risk	Low risk	High risk
Ou-yang et al. (2012)	Unclear	Low risk	Unclear	Unclear	Unclear	Unclear	Unclear	Low risk	Low risk	Unclear
Zhai et al. (2012)	Unclear	Low risk	Unclear	Unclear	Unclear	Unclear	Unclear	Low risk	Low risk	Unclear
Du and Chen (2013)	Unclear	Low risk	Unclear	Unclear	Unclear	Unclear	Unclear	Low risk	Low risk	High risk
Liu et al. (2013)	Unclear	Low risk	Unclear	Low risk	Unclear	Unclear	Unclear	Low risk	Low risk	High risk
Lin et al. (2014)	Low risk (random digital table method)	Low risk	Unclear	Unclear	Unclear	Unclear	Unclear	Low risk	Low risk	Unclear
Su et al. (2014)	Unclear	Low risk	Unclear	Unclear	Low risk	Unclear	Unclear	Low risk	Low risk	High risk
Zhou (2014)	Unclear	Low risk	Unclear	Unclear	Unclear	Unclear	Unclear	Low risk	Low risk	Unclear
Gao et al. (2015)	Unclear	Low risk	Unclear	Low risk	Unclear	Unclear	Unclear	Low risk	Low risk	High risk
Qian et al. (2015)	Unclear	Low risk	Unclear	Low risk	Unclear	Unclear	Unclear	Low risk	Low risk	High risk
Lu et al. (2016)	Unclear	Low risk	Unclear	Low risk	Unclear	Unclear	Unclear	Low risk	Low risk	High risk
Lu et al. (2017)	Unclear	Low risk	Unclear	Unclear	Unclear	Unclear	Unclear	Low risk	Low risk	Unclear
Pang et al. (2017)	Unclear	Low risk	Unclear	Unclear	Unclear	Unclear	Unclear	Low risk	Low risk	Unclear
Zhang et al. (2017)	Unclear	Low risk	Unclear	Unclear	Unclear	Unclear	Unclear	Low risk	Low risk	High risk
Shen et al. (2018)	Unclear	Low risk	Unclear	Unclear	Unclear	Unclear	Unclear	Low risk	Low risk	High risk
Zhou et al. (2018)	Unclear	Low risk	Unclear	Unclear	Unclear	Unclear	Unclear	Low risk	Low risk	High risk
Huang et al. (2019)	Unclear	Low risk	Unclear	Low risk	Unclear	Unclear	Unclear	Low risk	Low risk	Unclear
Liu et al. (2019)	Unclear	Low risk	Unclear	Unclear	Unclear	Unclear	Unclear	Low risk	Low risk	High risk
Luo et al. (2019)	Unclear	Low risk	Unclear	Unclear	Unclear	Unclear	Unclear	Low risk	Low risk	High risk
Wang (2019)	Unclear	Low risk	Unclear	Low risk	Unclear	Unclear	Unclear	Low risk	Low risk	Unclear
Liu et al. (2020)	Unclear	Low risk	Unclear	Low risk	Unclear	Unclear	Unclear	Low risk	Low risk	High risk
Liu et al. (2021)	Unclear	Low risk	Unclear	Unclear	Unclear	Unclear	Unclear	Low risk	Low risk	Unclear
Sun et al. (2022)	Unclear	Low risk	Unclear	Unclear	Unclear	Unclear	Unclear	Low risk	Low risk	High risk
Ma et al. (2022)	Unclear	Low risk	Unclear	Unclear	Unclear	Unclear	Unclear	Low risk	Low risk	Unclear
Zheng et al. (2022)	Unclear	Low risk	Unclear	Unclear	Unclear	Unclear	Unclear	Low risk	Low risk	High risk
Ding and Ren (2023)	Unclear	Low risk	Unclear	Unclear	Unclear	Unclear	Unclear	Low risk	Low risk	Unclear
Yu et al. (2023)	Unclear	Low risk	Unclear	Unclear	Unclear	Unclear	Unclear	Low risk	Low risk	High risk
Zhang et al. (2023)	Unclear	Low risk	Unclear	Low risk	Unclear	Unclear	Unclear	Low risk	Low risk	High risk

Note: ① whether the generation or application of the allocation sequence is sufficient; ② whether the baselines are the same; ③ whether allocation concealment is sufficient; ④ whether the animals were randomly placed during the study; ⑤ whether researchers and animal breeders were blinded; ⑥ whether random selection was conducted during result evaluation; ⑦ whether to use blinding methods for the evaluators of the results; ⑧ whether incomplete data were reported; ⑨ whether the research report is not related to selective reporting of results; ⑩ whether there is no other bias present.

#### 3.3.3 Cell study

Fifteen cellular studies related to *Monascus purpureus* Went were included. Seven studies focused on the identification and screening of *Monascus purpureus* Went bacterial strains using cell biology techniques. Six studies determined the efficacy of *Monascus purpureus* Went, including the synthesis and *in vitro* anticancer activity of *Monascus purpureus* Went derivatives (n = 1), the blood pressure-lowering mechanism of *Monascus purpureus* Went (n = 1), the effect of *Monascus purpureus* Went on the secretion of TNF-alpha by peripheral blood single-nucleated cells of ankylosing spondylitis patients (n = 1), the effect of erythrocytes on the cell growth and molecular mechanisms of HCT-116 cells (n = 1), the effect of *Monascus purpureus* Went on the proliferation of myocardial fibroblasts (n = 1), and the role of *Monascus purpureus* Went in inducing apoptosis and autophagy in human colon cancer cells (n = 1).

Two studies examined the physiological characteristics of *Monascus purpureus* Went and *Monascus purpureus* Went (n = 1) and the morphological characteristics of TCM *Monascus purpureus* Went (n = 1).

### 3.4 Clinical trial

A total of 28 clinical trials studied the effects of TCM *Monascus purpureus* Went and its related Chinese patent medicine and preparation, including double-armed trials (n = 18), single-armed trials (n = 6), three-armed trials (n = 3), and multiple-armed trials (n = 1). One study was set up with a blank control, and four were set up with a placebo. The mode of administration was oral. Patient sources included tertiary hospitals (n = 19), secondary hospitals (n = 7), and other sources (n = 2). Diseases included dyslipidemia (n = 22), carotid atherosclerotic plaque (n = 3), unstable angina (n = 2), abnormal liver function (n = 2), hyperglycemia (n = 1), type 2 diabetes (n = 1), uremia (n = 1), and fatty liver (n = 1).

Among them, 19 studies examined the effects of Chinese patent medicines and preparations related to *Monascus purpureus* Went*,* including Xuezhikang capsule (n = 6), Zhibituo capsule (n = 2), Shengqu *Monascus purpureus* Went capsule (n = 2), lipid-lowering *Monascus purpureus* Went micro-powder (n = 2), *Monascus purpureus* Went compound preparation (n = 1), compound *Monascus purpureus* Went capsule (n = 1), *Monascus purpureus* Went capsule for reducing sugar (n = 1), *Monascus purpureus* Went flavonoid tablet (n = 1), rattan *Monascus purpureus* Went soft capsule (n = 1), Danxi *Monascus purpureus* Went wine (n = 1), and *C. chinensis* Franch. *Monascus purpureus* Went medicine (n = 1); nine studies determined the clinical therapeutic effect of TCM *Monascus purpureus* Went.

Among them, the most commonly used dose of TCM *Monascus purpureus* Went was 6 g/dose, administered once daily, with a treatment course of 90 days; the most commonly used dose of Xuezhikang capsule was 2 capsules/dose, administered twice daily, and the course of treatment was 56 days; the most commonly used dose of Zhibituo capsule was 1.05 g/dose, administered thrice daily, with a treatment course of 60 or 90 days; the most commonly used dose of Shengqu *Monascus purpureus* Went capsule was 2 capsules/dose or 4 capsules/dose, and the course of treatment was 84 or 168 days; and the most commonly used dose of lipid-lowering *Monascus purpureus* Went micro-powder was 1 capsule/dose, administered thrice daily, with a treatment course of 50 or 180 days.

In terms of indicator domains, physical and chemical testing indicators were applied 28 times, symptom and sign indicators were applied 13 times, and TCM symptom/syndrome indicators were applied six times.

For counting indicators, clinical efficacy was applied 14 times, and TCM evidence efficacy was applied four times. Measurement indicators included lipid levels (n = 25), liver and kidney functions (n = 6), blood routine (n = 2), TCM evidence points (n = 2), blood glucose levels (n = 2), body mass index (n = 2), serum inflammatory factor (n = 2), electrocardiography (n = 1), SGA scores (n = 1), and carotid ultrasound (n = 1). The specific information on clinical trials is presented in Table 4.

**TABLE 4:** Information on clinical trials related to *Monascus purpureus* Went.

Inclusion of studies	Disease	Source of subjects (hospital level)	Enrollment time	Number of men	Number of women	Total quantity	Age-bracket	Average age	Method of administration	Intervention	Frequency	Course of treatment	Indicator domain	Counting indicator	Measuring indicator
Cui et al. (2002)	Hyperlipemia	III	August 2000–December 2000	52	38	90	Did not report	59	Profess to be convinced	Compound *Monascus purpureus* Went capsule (Chinese patent medicine) vs Western medicine	2 grains/time, 3 times/day; 1 grain/time, 1 time/day	30 days	Symptoms and signs; physical and chemical testing	①	③④⑤⑥
Yin et al. (2016)	Primary dyslipidemia	III	December 2013–December 2015	22	118	140	Did not report	58.73 ± 10.20	Profess to be convinced	*Monascus purpureus* Went compound preparation (Chinese medicine preparation)	1 time/day	30 days	TCM symptoms/syndromes; physical and chemical testing	Did not report	③④⑤⑥⑦
Zhang and He (2016)	Hyperlipemia	III	August 2014–January 2016	40	40	80	Did not report	Did not report	Profess to be convinced	Western medicine vs TCM *Monascus purpureus* Went + Western medicine	1.5 g/time, 3 times/day; 5 mg/time, 2 times/day	90 days	Symptoms and signs; physical and chemical testing	①	③④⑤⑥
Chen et al. (2021)	Hyperlipemia was associated with abnormal liver function	III	July 2018–December 2019	43	29	102	25–75	53.48 ± 10.30	Profess to be convinced	Basic treatment + TCM *Monascus purpureus* Went	1 time/day	56 days	TCM symptoms/syndromes; physical and chemical testing	①	③④⑤⑥⑦⑧⑨
Luo and Zhong (2020)	Dyslipidemia	II	July–December 2019	28	32	60	45–75	Did not report	Profess to be convinced	Western medicine vs TCM *Monascus purpureus* Went	1 time/day; 2 times/day	90 days	Physical and chemical testing	Did not report	③④⑤⑥⑩
Zhang et al. (2020)	Hyperlipemia	Secondary hospital	May 2016–May 2018	25	36	61	Did not report	Did not report	Profess to be convinced	TCM *Monascus purpureus* Went	6 g/day	56 days	Symptoms and signs; physical and chemical testing	①	③④⑤⑥
Zhao et al. (2018)	Hyperlipemia was associated with abnormal liver function	III	June 2017–March 2018	50	30	80	25–75	Did not report	Profess to be convinced	Basic treatment + TCM *Monascus purpureus* Went vs. basic treatment	6 g/day	90 days	Symptoms and signs; physical and chemical testing	①	③④⑤⑥⑧⑨
Wang and Li (2018)	Hyperlipemia	III	March 2017–September 2017	50	34	84	Did not report	Did not report	Profess to be convinced	Western medicine vs. TCM *Monascus purpureus* Went	10 mg/time, 3 time/day; 3/day	90 days	Symptoms and signs; physical and chemical testing	①	③⑤⑥
Shi et al. (2015)	Dyslipidemia	III	Did not report	25	3	28	80–92	85.6 ± 4.8	Profess to be convinced	TCM *Monascus purpureus* Went	6 g/time, 2 times/day	56 days	Physical and chemical testing	Did not report	③④⑤⑥⑪⑫
Wang et al. (2022)	Uremia and hyperlipemia	III	September 2021–June 2022	31	37	68	62–86	68.13 ± 12.53	Profess to be convinced	Basic treatment + Western medicine vs. basic treatment + TCM *Monascus purpureus* Went	10 mg/day, 1 time/day; 6 g/day	56 days	Physical and chemical testing	Did not report	③④⑤⑥⑬⑭⑮⑯
Wu and Luo (2013)	Carotid atherosclerotic plaque	III	January 2011–December 2012	Did not report	Did not report	60	Did not report	Did not report	Profess to be convinced	TCM *Monascus purpureus* Went vs. Western medicine vs. blank control	6 g/time, 2 times/day; 1 time/day	180 days	TCM symptoms/syndromes; physical and chemical testing	②	③④⑤⑥⑰⑱⑲
Liu et al. (1998)	Hyperlipemia	III	July 1995–July 1996	41	35	76	Did not report	61.2 ± 3.6	Profess to be convinced	Zhibituo capsule (Chinese patent medicine)	3 times/day	60 days	Symptoms and signs; physical and chemical testing	①	③⑥
Qiu et al. (1997)	Hyperlipemia	III	August 1995–February 1996	112	64	176	Did not report	Did not report	Profess to be convinced	Di’ao Zhibituo capsule (Chinese patent medicine) vs. Western medicine	3 times/day	90 days	Symptoms and signs; physical and chemical testing	①	③⑤⑥
Lu et al. (2012)	Hyperlipemia	III	May 2010–October 2011	28	32	60	18–75	Did not report	Profess to be convinced	Basic treatment + Xuezhikang capsule (Chinese patent medicine) vs. basic treatment + placebo	2 times/day	56 days	TCM symptoms/syndromes; physical and chemical testing	②	③④⑤⑥
Wang et al. (1995)	Hyperlipemia	III	Did not report	261	185	446	Did not report	Did not report	Profess to be convinced	Xuezhikang capsule (Chinese patent medicine) vs. placebo	2 times/day	56 days	Symptoms and signs; physical and chemical testing	①	③④⑤⑥
Zhu (2013)	Unstable angina	Secondary hospital	June 2009–November 2012	55	25	80	66–89	Did not report	Profess to be convinced	Basic treatment + Xuezhikang capsule (Chinese patent medicine) vs. basic treatment	2 grains/time, 2 times/day	60 days	Symptoms and signs; physical and chemical testing	①	⑳
Tang and Chang (2012)	Hyperlipemia	Secondary hospital	2009–2011	62	28	90	38–65	48	Profess to be convinced	Basic treatment + Xuezhikang capsule (Chinese patent medicine) vs. basic treatment	2 times/day	28 days	Physical and chemical testing	Did not report	③④⑤⑥
Huang and Zhang (2005)	Unstable angina	Secondary hospital	Did not report	61	18	79	Did not report	Did not report	Profess to be convinced	Basic treatment + Xuezhikang capsule (Chinese patent medicine) vs. basic treatment	2 times/day	3 days	Physical and chemical testing	Did not report	⑮⑱⑲
Zheng and Li (2015)	Hyperlipemia	III	July 2013–July 2014	23	19	42	39–70	Did not report	Profess to be convinced	Xuezhikang capsule (Chinese patent medicine) vs. Western medicine	0.5 g/time, 2 times/day; 1 grain/time, 1 time/day	50 days	Physical and chemical testing	Did not report	③④⑤⑥
Wang et al. (2016)	Carotid atherosclerotic plaque	III	January–December 2015	58	62	120	55–73	61.4 ± 7.7	Profess to be convinced	Shengqu *Monascus purpureus* Went capsule (Chinese patent medicine) vs. Western medicine	4 grains/time, 3 times/day; 20 mg/day	168 days	Physical and chemical testing	Did not report	③④⑤⑥
Wang et al. (2014)	Dyslipidemia	III	2012–2013	38	82	120	35–73	51.4 ± 7.7	Profess to be convinced	Shengqu *Monascus purpureus* Went capsule (Chinese patent medicine) vs. Shengqu *Monascus purpureus* Went capsule (Chinese patent medicine) vs. Western medicine	2 grains/time, 3 times/day; 10 mg/day	84 days	Physical and chemical testing	Did not report	③④⑤⑥
Xu et al. (2019)	Hyperlipemia	II	Did not report	43	71	114	Did not report	44.59 ± 12.32	Profess to be convinced	Sea-buckthorn (*Hippophae rhamnoides* L.) *Monascus purpureus* Went capsule (Chinese patent medicine) vs. placebo	2 grains/time, 1 time/day	90 days	Symptoms and signs; TCM symptoms/syndromes; physical and chemical testing	①②	③④⑤⑥
Cao et al. (2007)	Type 2 diabetes	III	September 2006–July 2007	14	16	30	28–70	55.2	Profess to be convinced	*Monascus purpureus* Went capsule for reducing sugar (Chinese patent medicine)	3 grains/time, 1 time/day	90 days	Symptoms and signs; physical and chemical testing	①	⑩
Chen and Liu (2015)	Dyslipidemia and hyperglycemia	II	March 2013–December 2014	Did not report	Did not report	70	Did not report	50.50 ± 4.20	Profess to be convinced	Danxi *Monascus purpureus* Went wine (Traditional Chinese medicine preparation)	50 mL, 150 mL, and 250 mL/time; 2 times/day	90 days	Physical and chemical testing	Did not report	③④⑤⑥⑩⑬
Liu et al. (2011)	Dyslipidemia was associated with carotid atherosclerotic plaque	Other sources	April 2005–April 2006	23	37	60	Did not report	Did not report	Profess to be convinced	Lipid-lowering *Monascus purpureus* Went micro powder (Chinese patent medicine 1) vs. Xuezhikang capsule (Chinese patent medicine 2) vs. Western medicine	1 grain/time, 2 times/day; 2 grains/time, 2 times/day; 1 tablet/time, 1 time/day	180 days	TCM symptoms/syndromes; physical and chemical testing	②	③④⑤⑥⑰
Wu et al. (2005)	Hyperlipemia	Other sources	Did not report	Did not report	Did not report	80	Did not report	Did not report	Profess to be convinced	Half dose of lipid-lowering *Monascus purpureus* Went micro powder (Chinese patent medicine 1) vs. lipid-lowering *Monascus purpureus* Went micro powder (Chinese patent medicine 2) vs. lipid-lowering *Monascus purpureus* Went crude powder (Chinese patent medicine 3) vs. Xuezhikang capsule (Chinese patent medicine 4)	1 grain/time, 2 times/day; 0.6 g/time, 2 times/day	50 days	Symptoms and signs; physical and chemical testing	①	③④⑤⑥⑪⑫
Yang et al. (2024)	Intervention in non-alcoholic fatty liver	III	October 2020–October 2021	41	39	80	32–60	44.52 ± 6.2	Profess to be convinced	Western medicine vs. *Coptis chinensis* Franch. *Monascus purpureus* Went medicine (Chinese medicine preparation) + Western medicine	3 times/day; 1 time/day	56 days	Physical and chemical testing	Did not report	③④⑤⑥⑧⑨
He et al. (2007)	Hyperlipemia	III	Did not report	69	21	100	18–65	44.06 ± 9.17	Profess to be convinced	*Monascus purpureus* Went Flavonoid tablet (Chinese patent medicine) vs. placebo	2.4 g/day	30 days	Symptoms and signs; physical and chemical testing	①	③⑤⑥

Note: ①, clinical efficacy; ②, efficacy of TCM syndrome; ③, total cholesterol (TC) level; ④, low-density lipoprotein cholesterol (LDL-C) level; ⑤, triglyceride (TG) level; ⑥, high-density lipoprotein cholesterol (HDL-C) level; ⑦, TCM syndrome points; ⑧, ALT horizontal; ⑨, AST horizontal; ⑩, blood glucose check; ⑪, ApoB level; ⑫, ApoA level; ⑬, body mass index (BMI); ⑭, serum albumin (ALB); ⑮, C-reactive protein (CRP) level; ⑯, SGA grade; ⑰, carotid artery ultrasound; ⑱, routine blood test; ⑲, liver and kidney function; ⑳, electrocardiogram.

A total of 21 studies were randomized controlled trials (79%), and six were self-controlled trials (21%). Among the 22 randomized controlled trials, only 15 reported “randomization”; six used a randomized numeric table method, and one used lottery method. Only one study performed allocation concealment, four studies blinded patients, and one study blinded outcome assessors. The remaining studies did not perform allocation concealment or apply blinding; no missing data were reported. Thirteen studies exhibited other sources of bias, such as not reporting the source of funding or trial enrollment, while nine studies provided complete reporting. The risk of bias assessment of clinical trials is presented in Table 5.

**TABLE 5:** Risk of bias assessment of RCTs.

Included in the study	Generation of the randomly assigned sequences	Allocation concealment	Subjects were blinded	Blinded to the outcome assessors	Resulting data integrity	Selective report	Other bias
Cui et al. (2002)	Unclear	Unclear	High risk	Unclear	Low risk	Low risk	High risk
Zhang and He (2016)	Unclear	Unclear	High risk	Unclear	Low risk	Low risk	High risk
Luo and Zhong (2020)	Low risk (random digital table method)	Unclear	High risk	Unclear	Low risk	Low risk	Low risk
Zhao et al. (2018)	Unclear	Unclear	High risk	Unclear	Low risk	Low risk	High risk
Wang and Li (2018)	Low risk (random digital table method)	Unclear	High risk	Unclear	Low risk	Low risk	Low risk
Wang et al. (2022)	Low risk (lottery method)	Unclear	High risk	Unclear	Low risk	Low risk	Low risk
Wu and Luo (2013)	Low risk (random digital table method)	Unclear	High risk	Unclear	Low risk	Low risk	High risk
Liu et al. (1998)	Unclear	Unclear	High risk	Unclear	Low risk	Low risk	High risk
Qiu et al. (1997)	Unclear	Unclear	High risk	Unclear	Low risk	Low risk	High risk
Lu et al. (2012)	Unclear	Unclear	Low risk	Unclear	Low risk	Low risk	High risk
Wang et al. (1995)	Unclear	Unclear	Low risk	Unclear	Low risk	Low risk	High risk
Zhu (2013)	Unclear	Unclear	High risk	Unclear	Low risk	Low risk	High risk
Huang and Zhang (2005)	Unclear	Unclear	High risk	Unclear	Low risk	Low risk	High risk
Zheng and Li (2015)	Unclear	Unclear	High risk	Unclear	Low risk	Low risk	High risk
Wang et al. (2016)	Unclear	Unclear	High risk	Unclear	Low risk	Low risk	Low risk
Wang et al. (2014)	Unclear	Unclear	High risk	Unclear	Low risk	Low risk	Low risk
Xu et al. (2019)	Unclear	Low risk	Low risk	Unclear	Low risk	Low risk	Low risk
Chen and Liu (2015)	Low risk (random digital table method)	Unclear	High risk	Unclear	Low risk	Low risk	High risk
Liu et al. (2011)	Low risk (random digital table method)	Unclear	High risk	Unclear	Low risk	Low risk	Low risk
Wu et al. (2005)	Unclear	Unclear	High risk	Unclear	Low risk	Low risk	Low risk
Yang et al. (2024)	Low risk (random digital table method)	Unclear	High risk	Unclear	Low risk	Low risk	Low risk
He et al. (2007)	Unclear	Unclear	Low risk	Low risk	Low risk	Low risk	High risk

## 4 Discussion and analysis

### 4.1 Content determination method of active ingredients in *Monascus purpureus* Went

#### 4.1.1 High-performance liquid chromatography

HPLC is the most commonly used method to determine the content of active ingredients in *Monascus purpureus* Went, particularly *lovastatin* and citrinin (*orange mold*) (Wang and Gao, 2006; Wen et al., 2011; Hao et al., 2017; Tan et al., 2015; Qiu et al., 2012; Luo et al., 2003; Zhang et al., 2016; Lu et al., 2019; Zhang et al., 1997; Liu, 2007; Fan, 2013; Zhang et al., 2001; Chen and Zhao, 2007; Zhu et al., 2023; Huang, 2000; Chen et al., 2008; Wang, 2014; Gao et al., 2022; Li et al., 2011; Wang, 2000; Song et al., 1999b; Xie et al., 2010; Huang et al., 2014; Wang et al., 2020; Li and Li, 2008; Lv et al., 2020; Li et al., 2010; Li et al., 2019; Qi et al., 2021), and is characterized by simplicity, accuracy, reliability, good repeatability, and high sensitivity.

#### 4.1.2 Thin-layer chromatography scan

TLCS is a method used to determine the content of phosphatidylcholine and daidzein in *Monascus purpureus* Went and Xuezhikang capsule (Xu et al., 2000; Xu et al., 2001), which is characterized by simplicity and good repeatability and can be used to control the quality of *Monascus purpureus* Went and its preparations.

#### 4.1.3 Capillary electrophoresis

CE is a method used to determine the contents of *lovastatin* and citrinin (a compound produced by *orange mold*) in *Monascus purpureus* Went (Chen et al., 2007a; Zhang et al., 2008), which is characterized by simplicity, speed, high sensitivity, and good repeatability in detecting certain charged components.

#### 4.1.4 Other methods

AA is a method used to determine the content of amino acids in *Monascus purpureus* Went (Chen et al., 2007b), which is characterized by high sensitivity and accuracy. GC is a method used to determine the contents of oleic acid and linoleic acid in *Monascus purpureus* Went (Zhang et al., 2010). QuEChERS-UPLC-MRM-IDA Criteria-EPI is a method used to determine and quantify the content of *lovastatin* (Wang, 2016). PSA, ASA, and DNS are methods used to determine the content of polysaccharides in *Monascus purpureus* Went (Fang et al., 2021). FAAS is a method used to determine the content of metal trace elements in *Monascus purpureus* Went (Lin et al., 2001). SPSS PCA and SPSS CA were used to analyze trace elements in *Monascus purpureus* Went (Cao and Wu, 2009) to reveal the relationships and distribution patterns between components.

### 4.2 Chemical ingredients of *Monascus purpureus* Went

A variety of active ingredients in *Monascus purpureus* Went provide the material basis for the pharmacological effect of *Monascus purpureus* Went, mainly including Monascus pigment, monacolin K, ergosterol, stigmasterol, *Monascus purpureus* Went polysaccharide, and a variety of enzymes.

#### 4.2.1 Monascus pigment

Monascus pigment is a secondary metabolite of *Monascus purpureus* Went*.* Monascus pigment not only provides a unique color for *Monascus purpureus* Went but also possesses physiological activities such as antioxidant, antibacterial, and anti-inflammatory properties. So far, as many as 54 types of Monascus pigment have been identified, among which the more intensively studied pigments include yellow Monascus pigment, ankaflavin, rubropunctamine, and monascorubramine. It has been demonstrated that yellow Monascus pigment has a protective effect on the liver of hyperlipemia mice and can regulate blood lipids, and the mechanism of action may be related to the regulation of lipid metabolism and activation of the AMP-activated protein kinase (AMPK) pathway to stimulate fatty acid oxidation (Fang et al., 2021). Monascorubramine can promote the apoptosis of gastric cancer AGS cells, while no obvious inhibitory effect on normal cells was observed, and its therapeutic coefficient is higher than that of paclitaxel, which is a conventional chemotherapeutic drug for gastric cancer (Lin et al., 2001). The safety of Monascus pigment has been proven to be high through acute and chronic toxicity studies, and it has been widely used as an additive ingredient of Monascus pigment in food and cosmetic production processes (Jiang et al., 2021; Xu et al., 2018; Pan et al., 2023).

#### 4.2.2 Statin ingredients

The statin component of *Monascus purpureus* Went has a wide range of applications in the field of medicine. In the late 1970s, Japanese scientists discovered and isolated a chemical component called monacolin K from the fermentation of *Monascus purpureus* Went, which can inhibit cholesterol synthesis in the body (Kong et al., 2005). Further studies revealed that the statin component in *Monascus purpureus* Went is similar to chemically synthesized statins in terms of its lipid-lowering effect. Among them, *lovastatin*, the most common statin component in *Monascus purpureus* Went, was formally approved by the FDA in the United States in 1987 and became the first generation of statin lipid-lowering drugs. In addition, *lovastatin* has an anti-tumor effect, which can induce the activation of the key molecule of apoptosis, caspase 7, and its receptor PARP protein cleavage. *Lovastatin* can inhibit the proliferation of PC3 cells and induce apoptosis in prostate cancer and has been shown to be efficacious in common tumors, such as gastric cancer, carcinoma of the bile duct, and nasopharyngeal carcinoma (NPC) (Xu et al., 2018).

#### 4.2.3 Sterol composition

*Monascus purpureus* Went produces a variety of sterol components during the fermentation process, such as ergosterol and stigmasterol. Ergosterol is one of the precursor substances of vitamin D2, which can be converted into vitamin D2 after ultraviolet irradiation, and is involved in the metabolism of calcium and phosphorus in the body, which has an obvious effect on the prevention and treatment of rickets in infants and young children and the promotion of calcium and phosphorus absorption in pregnant women and the elderly. Studies have shown that ergosterol can significantly reduce the blood glucose level of diabetic nephropathy model mice, providing a theoretical basis for ergosterol to be used in the clinical treatment of diabetic nephropathy (Xu et al., 2018). Soysterol can competitively inhibit the absorption of cholesterol in the human body and effectively reduce the level of serum cholesterol, which is an important active ingredient in regulating lipid balance and preventing cardiovascular and cerebrovascular diseases (Ge et al., 2012).

#### 4.2.4 Other ingredients

*Monascus purpureus* Went contains a variety of other active ingredients, such as *Monascus purpureus* Went polysaccharide, unsaturated fatty acids, a variety of enzymes (e.g., amylase, protease, and lipase), and flavonoids, which also play important roles in the pharmacological effects of *Monascus purpureus* Went.

For example, *Monascus purpureus* Went polysaccharides exhibit various physiological activities, such as immunoregulatory, anti-tumor, and lipid-lowering effects; unsaturated fatty acids help lower blood lipids and prevent cardiovascular diseases; and a variety of enzymes promote digestion and absorption of food in the human body.

### 4.3 Pharmacological mechanism of action of *Monascus purpureus* Went

#### 4.3.1 Lipid-lowering ability

*Monascus purpureus* Went has a lipid-lowering effect. This is mainly attributed to the enrichment of statins in *Monascus purpureus* Went, such as monacolin K, which is the active ingredient of *lovastatin.* A number of included clinical trials have shown (Yin et al., 2016; Zhang and He, 2016; Chen et al., 2021; Luo and Zhong, 2020; Zhang et al., 2020; Zhao et al., 2018; Shi et al., 2015; Gao et al., 2016) that *Monascus purpureus* Went has a lipid-lowering effect, generally reducing plasma total cholesterol (TC) levels, low-density lipoprotein (LDL) levels, and triglyceride (TG) levels and also increasing high-density lipoprotein (HDL) levels.

#### 4.3.2 Oxidation resistance

Antioxidant components such as polyphenolic compounds and flavonoids in *Monascus purpureus* Went can scavenge free radicals in the body and reduce cellular damage caused by oxidative stress. Studies have shown that the extracellular polysaccharides of *Monascus purpureus* Went have the ability to scavenge DPPH-free radicals, confirming the antioxidant effect of *Monascus purpureus* Went (Cai et al., 2010). This antioxidant effect helps slow down the cellular aging process and protects the cardiovascular system, the liver, and other organs from oxidative damage. In addition, the antioxidant effect of *Monascus purpureus* Went is complemented by its lipid-lowering effect, which works together to maintain the healthy state of the human body. Included clinical trials have shown that *Monascus purpureus* Went can reduce ALT and AST levels in patients with hyperlipemia and liver function abnormalities, thus protecting the liver.

#### 4.3.3 Anti-inflammatory action

*Monascus purpureus* Went has an anti-inflammatory effect, which is closely related to the various anti-inflammatory components it contains. Polyphenols and flavonoids in *Monascus purpureus* Went have antioxidant and free-radical scavenging ability, which can reduce the inflammatory response caused by oxidative stress. Studies have shown that *Monascus purpureus* Went can reduce serum TNF-α and CRP levels in inflammatory mouse models, confirming the anti-inflammatory effect of *Monascus purpureus* Went (Wang et al., 2006a; Wang et al., 2008), which makes *Monascus purpureus* Went potentially useful in the treatment of non-infectious inflammatory diseases, such as arthritis and dermatitis.

#### 4.3.4 Anti-tumor activity

In recent years, important progress has also been made in research on the anti-tumor effects of *Monascus purpureus* Went. *Monascus purpureus* Went polysaccharides and Monascus pigments in *Monascus purpureus* Went have an effect on inhibiting the growth and proliferation of tumor cells. These components affect the metabolism and signal transduction pathway of tumor cells through different pathways, thus exerting an anti-tumor effect. By determining the tumor inhibition rate, relative growth rate, and index of each organ in loaded mice, it was found that erythrocyte extracellular polysaccharides had a tumor-inhibitory effect on H22-loaded mice *in vivo* (Zhou et al., 2011). The determination of body weight, tumor weight, tumor suppression rate, and changes in spleen weight and spleen index of the loaded mice indicated that *Monascus purpureus* Went polysaccharides had a good inhibitory effect on tumor growth in loaded mice (Ding, 2007). Although the application of *Monascus purpureus* Went in anti-tumor therapy is still in the research stage, its potential should not be ignored.

### 4.4 Methodological quality

#### 4.4.1 Methodological quality of animal studies

The methodological quality assessment of animal studies related to *Monascus purpureus* Went found that most studies only reported “randomization,” while a few used the random number table. Assignment concealment and blinding of investigators or animal handlers and evaluators of results were not reported in most studies, which may have led to subjective bias in the expected experimental results, and only a few used assignment concealment and blinding. A number of studies did not report the randomization of the environment in which the animals were placed, which may have influenced objective environmental factors such as temperature, humidity, and light intensity in different locations on the experimental results. The result data were completely reported. In conclusion, it is necessary to improve the generation of random allocation sequences, allocation concealment, application of blinding, and randomness of the environment in animal studies related to *Monascus purpureus* Went.

#### 4.4.2 Methodological quality of clinical trials

The methodological quality assessment of clinical trials related to *Monascus purpureus* Went found that most trials only reported “randomization,” while a few used the random number table and lottery. Assignment concealment and blinding of patients and outcome evaluators were not reported in most trials, which may increase the risk of measurement bias and evaluator bias, and only a few used assignment concealment and blinding. Some trials did not report registration and conflict of interest, which could lead to inappropriate influence from sponsors. Outcome data were completely reported. In conclusion, it is necessary to improve the generation of random allocation sequences, allocation concealment, application of blinding, and reporting of funding sources in clinical trials related to *Monascus purpureus* Went.

#### 4.4.3 Suggestion for the study design

The common problems of methodological quality deficiencies in both animal studies and clinical trials are obvious, and the following suggestions are made: first, it is suggested that suitable random sequence generation methods, such as the random number table method, should be used to ensure the randomness and fairness of the allocation process. Second, it is suggested that allocation concealment and application of the double-blind method, including blinding of operators and observers, should be implemented. The person in charge of the operation should not know which group each patient is assigned to, in order to eliminate operator-induced subjective bias in the results. The person responsible for data collection and analysis should also not know the specific group to which each patient is assigned. The process of data collection and analysis should be carried out independently to ensure the objectivity and accuracy of the results. In addition, it is suggested that other conditions (e.g., environment and operation) should be strictly controlled for consistency during the study to ensure the reliability and repeatability of results. Finally, during data analysis, care should be taken to control the effect of confounding factors and other biases and to report in detail on the source of funding and registration to ensure the objectivity and accuracy of the results.

### 4.5 Limitation

There are some limitations to this review. First, the English-language literature obtained from the search was relatively limited. Second, the studies mainly focus on molecular and animal studies, with relatively few clinical trials. Third, some of the studies are of poor quality, and the reference value of their results may therefore be limited. Fourth, excluding studies for which the full texts could not be obtained may lead to data bias. Fifth, some of the clinical trials contained partially unreported information about the subjects, such as the patient source, enrollment time, and age range, which may reduce the strictness of the study. Sixth, only two meta-analyses were obtained and assessed for methodological quality and reporting standards in this study, and the results showed that they were of low quality, but due to their small number, these studies were not examined and described in detail in order to avoid study bias. The assessment of methodological quality and reporting standards for meta-analyses are presented in Supplementary Adds S2, S3.

### 4.6 Research implications

#### 4.6.1 Clinical safety

Existing studies have shown that the contraindications for *Monascus purpureus* Went productions mainly include the following: ① patients allergic to monacolin K/lovastatin or any excipients; ② patients with acute liver disease; ③ patients with severe renal impairment (eGFR <30 mL/min); ④ patients with various myopathies; and ⑤ pregnant women, lactating women, and women of childbearing age who have not taken effective contraceptive measures (Banach et al., 2022). It is recommended that its importance be emphasized in clinical practice.

#### 4.6.2 Fundamental research challenges

In terms of the bioactive ingredient biosynthetic pathways and regulatory mechanisms of substances produced during *Monascus* fermentation, the current challenges in the development of *Monascus purpureus* Went include improving the content of active ingredients like lovastatin and controlling the content of toxic metabolites like citrinin through methods such as optimizing fermentation parameters, mutagenic breeding, and genetic engineering.

#### 4.6.3 Other suggestions

More contamination risks derive from raw materials, microbial metabolism, or processing errors; the suggestions for the production quality control of *Monascus purpureus* Went are as follows: first, the quality control of raw materials should be optimized, the standardized management of fermentation strains should be strengthened, and high-quality fermentation strains should be accurately identified and screened. Second, the quality detection standard system should be improved, the specifications for the determination of active ingredient contents should be clarified, and the detection scope of safety indicators should be expanded. Third, the production process system must be optimized, the operation process must be standardized, fermentation parameters must be improved, and fermentation conditions must be strictly controlled. Fourth, the construction of the regulatory system must be improved, mandatory third-party safety reviews must be implemented, and the post-market supervision of products must be strengthened.

To promote the high-quality development of *Monascus purpureus* Went studies, the suggestions are as follows: first, standards should be set up. Unified quality standards for experimental design and efficacy evaluation should be developed to improve comparability between different studies. The production process and quality control of *Monascus purpureus* Went should be standardized to ensure the stable quality of *Monascus purpureus* Went products used in research and application. Second, multidisciplinary cooperation should be promoted. Experts in the fields of pharmacy, medicine, biology, and other multidisciplinary fields should be encouraged to cooperate in the studies and explore the pharmacological action mechanism of *Monascus purpureus* Went in depth from different perspectives. Third, the research and development of new drugs should be encouraged. Modern preparation technology should be actively used to develop new *Monascus purpureus* Went preparations with more stable efficacy. Fourth, more research on the combination of *Monascus purpureus* Went and other drugs should be encouraged to observe the therapeutic effect and safety. Fifth, human pharmacokinetic studies and clinical trials should be performed to explore the effective dosage range of *Monascus purpureus* Went products, validate safety and prevent adverse events, and provide evidentiary support for clinical practice. Concurrently, key procedures, including ethical review and informed consent, must be strictly implemented throughout the process.

## 5 Conclusion

As a type of traditional Chinese medicine, the pharmacological action mechanism of *Monascus purpureus* Went is extensive and complex. Given the extensive global use of Monascus purpureus Went products, we have gradually revealed its mechanism of action in regulating blood lipids and exerting anti-inflammatory, anti-oxidant, and anti-tumor effects through modern science research and technology. Moreover, we also need to pay attention to the contraindications and safety issues associated with the use of *Monascus purpureus* Went to ensure its safety and effectiveness in our daily lives.

## Data Availability

The original contributions presented in the study are included in the article/Supplementary Material; further inquiries can be directed to the corresponding authors.

## Abbreviations

AA, automatic amino acid analyzer; AMPK, AMP-activated protein kinase; ASA, anthrone–sulfuric acid; CE, capillary electrophoresis; CNKI, China National Knowledge Infrastructure; DNS, dinitrosalicylic acid; FAAS, flame atomic absorption spectroscopy; GC, gas chromatography; HDL, high-density lipoprotein; HPLC, high-performance liquid chromatography; LDL, low-density lipoprotein; NPC, nasopharyngeal carcinoma; PSA, phenol–sulfuric acid; QuEChERS-UPLC-MRM-IDA Criteria-EPI; quick, easy, cheap, effective, rugged, safe–ultra-performance liquid chromatography–multiple reaction monitoring–ion-dependent acquisition–criteria-enhanced product ion; RAPD, random amplified polymorphic DNA; SMF, secondary metabolite fingerprint; SPSS CA, statistical package for the social sciences cluster analysis; SPSS PCA, statistical package for the social sciences principal component analysis; TC, total cholesterol; TCM, traditional Chinese medicine; TG, triglyceride; TLCS, thin-layer chromatography scan.
