# Supplementary material for: The pharmacology and mechanism of action of Monascus purpureus Went: a scoping review
Source: Front Pharmacol. 2025 Jul 30;16:1600460. doi: 10.3389/fphar.2025.1600460 (PMC12343590; doi:10.3389/fphar.2025.1600460)
Supplement: Supplementary file 1 [file Table1.docx]

**Add 1. The search terms of all databases**

China National Knowledge Infrastructure(CNKI): (SU=('红曲 ' OR '红曲霉')) AND (TKA=('药理机制' OR '药理作用' OR '生物活性' OR '活性成分'))

Wanfang database: 主题: ("红曲" OR "红曲霉") 与 (题名或关键词: ("药理机制" OR "药理作用" OR "生物活性" OR "活性成分"))

VIP database: (U=红曲 OR U=红曲霉) AND (M=药理机制 OR M=药理作用 OR M=生物活性 OR M=活性成分)

SinoMed: ("红曲"[题目: 智能] OR "红曲霉"[题目: 智能] AND ("药理机制"[摘要: 智能] OR "药理作用"[摘要: 智能] OR "生物活性"[摘要: 智能] OR "活性成分"[摘要: 智能]

PubMed: ((Monascus [Title/Abstract]) OR (red yeast [Title/Abstract]) OR (Monascus purpureus [Title/Abstract])) AND ((pharmacology [Text Word]) OR (pharmacological effect [Text Word]) OR (pharmacological mechanism [Text Word]) OR (biological activity [Text Word]) OR (active ingredient [Text Word]))

**Add 2. Methodological quality assessment of Meta-analysis (AMSTAR-2)：**

|  | Item | Han Zhuojun 2022^[143]^ | Cai Le 2017^[144]^ |
| --- | --- | --- | --- |
| 1 | Did the research questions and inclusion criteria for the review include the components of PICO? | Yes | No |
| 2 | Did the report of the review contain an explicit statement that the review methods were established prior to the conduct of the review and did the report justify any significant deviations from the protocol? | Partial yes | Partial yes |
| 3 | Did the review authors explain their selection of the study designs for inclusion in the review? | Yes | Yes |
| 4 | Did the review authors use a comprehensive literature search strategy? | Partial yes | Partial yes |
| 5 | Did the review authors perform study selection in duplicate? | No | No |
| 6 | Did the review authors perform data extraction in duplicate? | No | No |
| 7 | Did the review authors provide a list of excluded studies and justify the exclusions? | No | No |
| 8 | Did the review authors describe the included studies in adequate detail? | Partial yes | No |
| 9 | Did the review authors use a satisfactory technique for assessing the risk of bias (RoB) in individual studies that were included in the review? | Yes | No |
| 10 | Did the review authors report on the sources of funding for the studies included in the review? | No | No |
| 11 | If meta-analysis was performed, did the review authors use appropriate methods for statistical combination of results? | Yes | Yes |
| 12 | If meta-analysis was performed, did the review authors assess the potential impact of RoB in individual studies on the results of the meta-analysis or other evidence synthesis? | Yes | No |
| 13 | Did the review authors account for RoB in primary studies when interpreting/discussing the results of the review? | Yes | No |
| 14 | Did the review authors provide a satisfactory explanation for, and discussion of, any heterogeneity observed in the results of the review? | Yes | No |
| 15 | If they performed quantitative synthesis did the review authors carry out an adequate investigation of publication bias (small study bias) and discuss its likely impact on the results of the review? | No | No |
| 16 | Did the review authors report any potential sources of conflict of interest, including any funding they received for conducting the review? | No | No |

**Add 3. Reporting standard assessment of Meta-analysis (PRISMA 2020)**

| Section and topic | Item | Checklist item | Han Zhuojun 2022^[143]^ | Cai Le 2017^[144]^ |
| --- | --- | --- | --- | --- |
| **Title** |  |  |  |  |
| Title | 1 | Identify the report as a systematic review. | Complete report | Complete report |
| **Abstract** | | | |  |
| Abstract | 2 | [See the PRISMA 2020 for Abstracts checklist.](https://systematicreviewsjournal.biomedcentral.com/articles/10.1186/s13643-021-01626-4#Tab2) | Partial report | Partial report |
| **Introduction** | | | |  |
| Rationale | 3 | Describe the rationale for the review in the context of existing knowledge. | Complete report | Complete report |
| Objectives | 4 | Provide an explicit statement of the objective(s) or question(s) the review addresses. | Complete report | Complete report |
| **Methods** | | | |  |
| Eligibility criteria | 5 | Specify the inclusion and exclusion criteria for the review and how studies were grouped for the syntheses. | Complete report | Complete report |
| Information sources | 6 | Specify all databases, registers, websites, organizations, reference lists and other sources searched or consulted to identify studies. Specify the date when each source was last searched or consulted. | Partial report | Partial report |
| Search strategy | 7 | Present the full search strategies for all databases, registers and websites, including any filters and limits used. | Complete report | Complete report |
| Selection process | 8 | Specify the methods used to decide whether a study met the inclusion criteria of the review, including how many reviewers screened each record and each report retrieved, whether they worked independently, and if applicable, details of automation tools used in the process. | Not reported | Not reported |
| Data collection process | 9 | Specify the methods used to collect data from reports, including how many reviewers collected data from each report, whether they worked independently, any processes for obtaining or confirming data from study investigators, and if applicable, details of automation tools used in the process. | Partial report | Not reported |
| Data items | 10a | List and define all outcomes for which data were sought. Specify whether all results that were compatible with each outcome domain in each study were sought (e.g. for all measures, time points, analyses), and if not, the methods used to decide which results to collect. | Complete report | Complete report |
|  | 10b | List and define all other variables for which data were sought (e.g. participant and intervention characteristics, funding sources). Describe any assumptions made about any missing or unclear information. | Partial report | Not reported |
| Study risk of bias assessment | 11 | Specify the methods used to assess risk of bias in the included studies, including details of the tool(s) used, how many reviewers assessed each study and whether they worked independently, and if applicable, details of automation tools used in the process. | Partial report | Not reported |
| Effect measures | 12 | Specify for each outcome the effect measure(s) (e.g. risk ratio, mean difference) used in the synthesis or presentation of results. | Complete report | Not reported |
| Synthesis Methods | 13a | Describe the processes used to decide which studies were eligible for each synthesis (e.g. tabulating the study intervention characteristics and comparing against the planned groups for each synthesis (item #5)). | Complete report | Complete report |
|  | 13b | Describe any methods required to prepare the data for presentation or synthesis, such as handling of missing summary statistics, or data conversions. | Complete report | Not reported |
|  | 13c | Describe any methods used to tabulate or visually display results of individual studies and syntheses. | Complete report | Complete report |
|  | 13d | Describe any methods used to synthesise results and provide a rationale for the choice(s). If meta-analysis was performed, describe the model(s), method(s) to identify the presence and extent of statistical heterogeneity, and software package(s) used. | Complete report | Not reported |
|  | 13e | Describe any methods used to explore possible causes of heterogeneity among study results (e.g. subgroup analysis, meta-regression). | Complete report | Not reported |
|  | 13f | Describe any sensitivity analyses conducted to assess robustness of the synthesized results. | Complete report | Not reported |
| Reporting bias assessment | 14 | Describe any methods used to assess risk of bias due to missing results in a synthesis (arising from reporting biases). | Complete report | Not reported |
| Certainty assessment | 15 | Describe any methods used to assess certainty (or confidence) in the body of evidence for an outcome. | Not reported | Not reported |
| **Results** | | | |  |
| Study selection | 16a | [Describe the results of the search and selection process, from the number of records identified in the search to the number of studies included in the review, ideally using a flow diagram](https://systematicreviewsjournal.biomedcentral.com/articles/10.1186/s13643-021-01626-4#Fig1) | Complete report | Not reported |
|  | 16b | Cite studies that might appear to meet the inclusion criteria, but which were excluded, and explain why they were excluded. | Complete report | Not reported |
| Study characteristics | 17 | Cite each included study and present its characteristics. | Complete report | Complete report |
| Risk of bias in studies | 18 | Present assessments of risk of bias for each included study. | Not reported | Not reported |
| Results of individual studies | 19 | For all outcomes, present, for each study: (a) summary statistics for each group (where appropriate) and (b) an effect estimate and its precision (e.g. confidence/credible interval), ideally using structured tables or plots. | Complete report | Complete report |
| Results of syntheses | 20a | For each synthesis, briefly summarize the characteristics and risk of bias among contributing studies. | Not reported | Not reported |
|  | 20b | Present results of all statistical syntheses conducted. If meta-analysis was done, present for each the summary estimate and its precision (e.g. confidence/credible interval) and measures of statistical heterogeneity. If comparing groups, describe the direction of the effect. | Not reported | Not reported |
|  | 20c | Present results of all investigations of possible causes of heterogeneity among study results. | Not reported | Not reported |
|  | 20d | Present results of all sensitivity analyses conducted to assess the robustness of the synthesised results. | Not reported | Not reported |
| Reporting biases | 21 | Present assessments of risk of bias due to missing results (arising from reporting biases) for each synthesis assessed. | Not reported | Not reported |
| Certainty of evidence | 22 | Present assessments of certainty (or confidence) in the body of evidence for each outcome assessed. | Not reported | Not reported |
| **Discussion** | | | |  |
| Discussion | 23a | Provide a general interpretation of the results in the context of other evidence. | Complete report | Complete report |
|  | 23b | Discuss any limitations of the evidence included in the review. | Complete report | Not reported |
|  | 23c | Discuss any limitations of the review processes used. | Complete report | Not reported |
|  | 23d | Discuss implications of the results for practice, policy, and future research. | Complete report | Complete report |
| **Other information** | | | |  |
| Registration and protocol | 24a | Provide registration information for the review, including register name and registration number, or state that the review was not registered. | Not reported | Not reported |
|  | 24b | Indicate where the review protocol can be accessed, or state that a protocol was not prepared. | Not reported | Not reported |
|  | 24c | Describe and explain any amendments to information provided at registration or in the protocol. | Not reported | Not reported |
| Support | 25 | Describe sources of financial or non-financial support for the review, and the role of the sponsors in the review. | Complete report | Not reported |
| Competing interests | 26 | Declare any competing interests of review authors. | Not reported | Not reported |
| Availability of data, code, and other materials | 27 | Report which of the following are publicly available and where they can be found: template data collection forms; data extracted from included studies; data used for all analyses; analytic code; any other materials used in the review. | Not reported | Not reported |

**Add 4. Statistics for included reviews topics:**

①Clinical efficacy; ②Pharmacologic mechanism; ③Active ingredient; ④Safety evaluation; ⑤Classification criteria; ⑥*Monascus purpureus* Went-related food and drug use; ⑦Origin; ⑧Production; ⑨Selective breeding; ⑩Industry standard

| Topic | Included reviews |
| --- | --- |
| Clinical efficacy(11) | Han Zhuojun 2022^[143]^ |
|  | Cicero AFG 2023^[145]^ |
|  | Cai Ruozhou 2014^[146]^ |
|  | Xia Linghong 2018^[147]^ |
|  | Yuan Tingting 2010^[148]^ |
|  | Jiang Weimin 2010^[149]^ |
|  | Shi kuo 2015^[150]^ |
|  | Yin Mengmei 2017^[151]^ |
|  | Xu Guicheng 2008^[152]^ |
|  | Bi Liming 2013^[153]^ |
|  | Xia Yishi 2018^[154]^ |
| Pharmacologic mechanism(24) | Xing Wangxing 2006^[155]^ |
|  | Yang Dong 2007^[156]^ |
|  | Buzzelli L 2024^[157]^ |
|  | Wei Ruonan 2023^[6]^ |
|  | Song Hongtao 1999^[2]^ |
|  | Xie Zhongmeng 1996^[7]^ |
|  | Kong Yanling 2005^[11]^ |
|  | Wang Jifan, 2013^[158]^ |
|  | Zhou Xiangzhen 2016^[159]^ |
|  | Wang Qiong 2022^[160]^ |
|  | Chen Yiguang 2000^[1]^ |
|  | Ji Yuanzhong 2005^[161]^ |
|  | Fu Jinquan 2002^[162]^ |
|  | Jiang Yuanji Qi 2021^[137]^ |
|  | Zhang Mulan 2022^[163]^ |
|  | Cai Jieyun 2010^[164]^ |
|  | Wen Xingyu 2018^[165]^ |
|  | Feng Yingnan 2020^[166]^ |
|  | Sun Pengyi 2002^[167]^ |
|  | Anonymous name 2020^[168]^ |
|  | Hu Xiangguo 2017^[169]^ |
|  | Gao Hong 1999^[170]^ |
|  | Li Zhongqing 1997^[171]^ |
|  | Zhou Yufang 2000^[172]^ |
| Active ingredient(19) | Li Xuemei 2011^[173]^ |
|  | Cheng Xiaoxia 2011^[174]^ |
|  | Xu Shisheng 2018^[138]^ |
|  | Wen Xuewei 2011^[175]^ |
|  | Liu Xi, 2006^[176]^ |
|  | Du Jun 2003^[177]^ |
|  | Zhao Hai 2002^[178]^ |
|  | Mu Qi 2018^[179]^ |
|  | Pan Ronghua 2023^[139]^ |
|  | Changhua 2001^[180]^ |
|  | Li Xuemei 2011^[181]^ |
|  | Jiang Lixiang 2011^[182]^ |
|  | Ma Zubing 2017^[183]^ |
|  | Wen Jing 2001^[9]^ |
|  | Dai Chunhua 1999^[184]^ |
|  | Chen Ping 2009^[185]^ |
|  | Chen Shan 2015^[186]^ |
|  | Anonymous name 2010^[187]^ |
|  | Zhang M 2024^[188]^ |
| Safety evaluation(3) | Cai Le 2017^[144]^ |
|  | Chen Qingsen 1998^[189]^ |
|  | Wang Huili 2010^[190]^ |
| Classification criteria(1) | Su Guotong 2004^[191]^ |
| *Monascus purpureus* Went-related food and drug use(4) | Zhang Yingjie 2011^[192]^ |
|  | Pang Huixin 2016^[193]^ |
|  | Xie Lei 2019^[194]^ |
|  | Zhuo Linxia 2012^[195]^ |
| Origin(4) | Li Dong 2007^[196]^ |
|  | Xing Wangxing 2000^[197]^ |
|  | Lin Feng 2017^[198]^ |
|  | Li Dong 2009^[199]^ |
| Production (1) | Gaowanshan 1988^[200]^ |
| Selective breeding(2) | Lin Feng 2018^[201]^ |
|  | Li P 2024^[202]^ |
| Industry standard (1) | Jiang Bingjie 2015^[203]^ |
